# SIRT1 and SIRT2 Activity Control in Neurodegenerative Diseases

**DOI:** 10.3389/fphar.2020.585821

**Published:** 2021-01-12

**Authors:** Ramu Manjula, Kumari Anuja, Francisco J. Alcain

**Affiliations:** ^1^ Department of Pharmacology, Yale School of Medicine, New Haven, CT, United States; ^2^ School of Biotechnology, KIIT University, Bhubaneswar, India; ^3^ Department of Medical Sciences, Faculty of Medicine, University of Castilla-La Mancha, Albacete, Spain; ^4^ Oxidative Stress and Neurodegeneration Group, Regional Center for Biomedical Research, University of Castilla-La Mancha, Ciudad Real, Spain

**Keywords:** neurodegenerative diseases, SIRT1, SIRT2, modulators, neuroprotective mechanism, resveratrol, selective pockets, sir reals

## Abstract

Sirtuins are NAD^+^ dependent histone deacetylases (HDAC) that play a pivotal role in neuroprotection and cellular senescence. SIRT1-7 are different homologs from sirtuins. They play a prominent role in many aspects of physiology and regulate crucial proteins. Modulation of sirtuins can thus be utilized as a therapeutic target for metabolic disorders. Neurological diseases have distinct clinical manifestations but are mainly age-associated and due to loss of protein homeostasis. Sirtuins mediate several life extension pathways and brain functions that may allow therapeutic intervention for age-related diseases. There is compelling evidence to support the fact that SIRT1 and SIRT2 are shuttled between the nucleus and cytoplasm and perform context-dependent functions in neurodegenerative diseases including Alzheimer’s disease (AD), Parkinson’s disease (PD), and Huntington’s disease (HD). In this review, we highlight the regulation of SIRT1 and SIRT2 in various neurological diseases. This study explores the various modulators that regulate the activity of SIRT1 and SIRT2, which may further assist in the treatment of neurodegenerative disease. Moreover, we analyze the structure and function of various small molecules that have potential significance in modulating sirtuins, as well as the technologies that advance the targeted therapy of neurodegenerative disease.

## Introduction

Sirtuins (Silent information regulator proteins) are essential anti-aging factors that are conserved in all kingdoms of living organisms, from bacteria to the human they have nearly identical structures and catalytic functions (Vassilopoulos et al., 2011). The sirtuin family is a NAD^+^ dependent histone deacetylase (HDAC) and is involved in the regulation of various activities such as deacetylation, ribosyltransferase (Michan and Sinclair, 2007), demalonylase, and desuccinylase (Taylor et al., 2008). The seven mammalian homologs of Sirtuins (SIRT1-7) are located in the different compartments of cells. They are involved in the regulation of various enzymes, which in turn, modulate crucial proteins involved in DNA repair, cell cycle and development, cellular senescence, and neuroprotection (Haigis and Sinclair, 2010; van de Ven et al., 2017). Therefore, they can be used as a therapeutic target for metabolic disorders and diseases.

All the isoforms of sirtuins can deacetylate both histone and non-histone proteins. SIRT1, SIRT6, and SIRT7 are localized in the nucleus and are involved in histone deacetylation and influence specific transcription factors to regulate cell cycle (Dryden et al., 2003; Michishita et al., 2005). SIRT2 is predominately present in the cytosol where it deacetylates non-histone proteins and is proactively shuttled to the nucleus and cytoplasm (North and Verdin, 2007). The localization of SIRT1 also changes conditionally depending upon cell type (Tanno et al., 2007). SIRT3, -4, -5 are located in the mitochondria and have been reported to regulate oxidative stress (Verdin et al., 2010). In yeast, these Sir2 proteins were found to increase longevity by acting on extrachromosomal rDNA circles and are suggested to be closest to SIRT1 in terms of structure and catalytic activity (Sinclair and Guarente, 1997; Ha and Huh, 2011). According to several studies, sirtuins were also shown to slow down the aging process in mammals and increased the lifespan by caloric restriction (CR) and SIRT1 is mainly involved in cell survival during caloric restriction (Cohen et al., 2004; Bishop and Guarente, 2007). SIRT3–5 expression favors mitochondrial oxidative metabolism, which induces stress tolerance during CR. Sirtuins also show pro-survival functions by blocking apoptosis and increasing cell survival through the regulation of forkhead transcription factors (FOXO) (Michan and Sinclair, 2007). Through these functions, sirtuins modulate the course of aging and affect neurodegenerative diseases, and SIRT activators/modulators could have therapeutic potential for neurodegenerative diseases (Jęśko et al., 2017).

**FIGURE 1:**
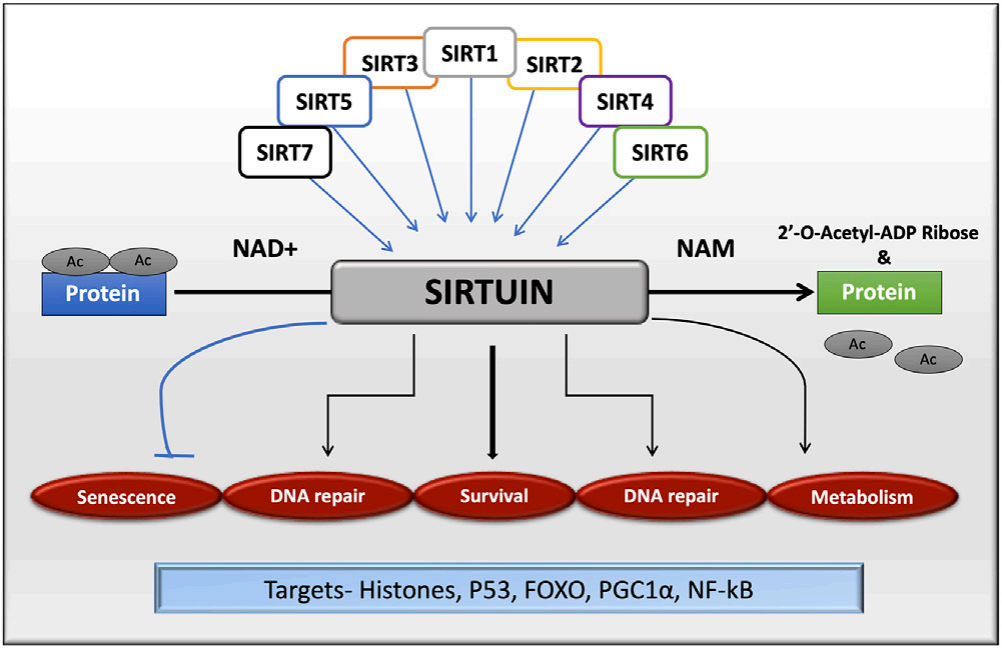
Sirtuin family in cellular functions by targeting histone and non-histone proteins. All the isoforms of sirtuins can deacetylate substrates using NAD^+^ as a cofactor, which is released as nicotinamide, a by-product during the reaction. Sirtuins are mainly involved in cell senescence, DNA repair, cell survival and metabolism.

Although neurodegenerative diseases are predominantly cell-type-specific, the various primary pathogenic progressions, which increase during aging, including protein misfolding, oxidative stress, cytoskeletal aberrations, disruption of calcium homeostasis, and inflammation are similar (Bossy-Wetze et al., 2004). A better understanding of a related mechanism may improve the possibility of therapeutic interventions against various neurodegenerative diseases. Among a plethora of treatments, sirtuins are grabbing attention as a target for many metabolic and neurodegenerative diseases. Among all the sirtuins, SIRT1 is the most studied and best understood mammalian sirtuin in terms of its function, activity, and regulation in diverse cellular processes. SIRT1 deacetylates histones such as H1-Lys26, H3-Lys9, Lys14, and H4-Lys16, and reduces methylation of histone H3-Lys79 (Imai et al., 2000; Vaquero et al., 2004) for the modulation of epigenetic information. SIRT1 deacetylates non-histone proteins which are associated with stress-related responses such as P53 (Vaziri et al., 2001), peroxisome proliferator-activated receptor gamma (PPAR-γ) (Wang et al., 2013), PPAR-γ coactivator-1 alpha (PGC-1α) (Gurd, 2011; Rodgers et al., 2008), FOXO3a, FOXO1, and FOXO4 thereby increasing mitochondrial biogenesis and cell’s resistance toward oxidative stress (Figure 1; Motta et al., 2004).

SIRT1 regulates cell proliferation, differentiation, survival, inflammation, and apoptosis by regulating NF-κB (Karin and Lin, 2002) and the members of the basic leucine zipper (bZIP) family proteins such as c-Fos and c-Jun. As a key regulator of metabolism, SIRT1 stimulates the fat and cholesterol catabolism and also regulates signaling proteins (Silva and Wahlestedt, 2010). Moreover, SIRT1 controls the gluconeogenic/glycolytic pathways through the transcriptional co-activator PGC-1α, which leads to an increase in the mitochondrial mass and function (Nemoto et al., 2005a). In mammals, SIRT1 and SIRT2 regulate the stability of the oncogenic protein c-Myc, enhancing activation of its transcription targets (Menssen et al., 2012). SIRT2 deacetylates *a*-tubulin during the cell cycle in the cytoplasm (North et al., 2003). However, when it is shuttled to the nucleus, it acts on many nuclear substrates such as p53, FOXO1, FOXO3a, histone H4, histone H3, and p300 (Black et al., 2008; Jin et al., 2008; Das et al., 2009).

Many studies have concluded that SIRT2 is mainly involved in mitotic checkpoints and centrosome integrity (Inoue et al., 2009; Inoue et al., 2007). SIRT2 is the major isoform which is expressed highly in brain cells mainly in myelin cells and also in olfactory and hippocampal neurons (Southwood et al., 2007; Pandithage et al., 2008), where it is involved in the suppression of glioma migration, cytoskeletal growth cone dynamics, neurite outgrowth and oligodendrocyte arborization (Harting and Knöll, 2010). SIRT2 inhibits colony formation in glioma cell lines (Hiratsuka et al., 2003) and arrests chromosomal instability by getting down-regulated. SIRT2 is associated with neurodegenerative diseases, and its inhibition delays the progression of the disease. SIRT2 has been reported to prevent α synuclein-mediated toxicity in PD by deacetylating lysine 6 and 10 of α-synuclein preventing its aggregation and toxicity (Outeiro et al., 2007; de Oliveira et al., 2017).

All these studies prove that mammalian sirtuins are involved in cancer, metabolic disorders, and neurological diseases. In recent years, numerous studies have led to the development of various modulators that either activate or inhibit the activity of sirtuins and thereby act as therapeutically potential drug molecules. Though there is a plethora of structure-function data available for SIRT1/2, their exact mechanisms of activity in cellular pathways are not yet known. Table 1 lists the best-known modulators of SIRT 1/2, which have been studied for their potential therapeutic effects in AD, PD, and HD.

**TABLE 1:** List of known sirtuin modulators.

Compound	The biological effect in NDD	Enzyme activity	Target/Specificity	References
Resveratrol	AD, PD, HD	SIRT1 EC_1.5_ = 46.2 μM	SIRT1 activator	Borra et al. (2005), Milne et al. (2007), Rocha-González et al. (2008), Turner et al. (2015)
Piceatannol	AD	—	SIRT1 activator	Kim et al. (2008)
SRT1720 (paeonol)	HD	SIRT1 EC50 = 0.16 μM	SIRT1 activator	Milne et al. (2007)
SRT2104	HD	—	SIRT1 activator	Libri et al. (2012), Jiang et al. (2014)
Nicotinamide (NAM)	AD, PD, HD	SIRT1 IC50 = 1.2 μM SIRT2 IC50 = 85 μM	Inhibitor- SIRT1-7	Avalos et al. (2005), Hathorn et al. (2011), Di Fruscia et al. (2015)
Sirtinol	—	ySir2 IC50 = 70 μM SIRT1 IC50 = 131 μM SIRT2 IC50 = 49 μM	Inhibitor- SIRT1 and 2	Shin et al. (2013), Li et al. (2014)
EX527/Selisistat	HD	SIRT1 IC50 = 0.098 μM SIRT2 IC50 = 19.6 μM	SIRT1 inhibitor	Napper et al. (2007), Süssmuth et al. (2015)
CHIC35	—	SIRT1 IC50 = 0.124 μM SIRT2 IC50 = 2.77 μM	Inhibitor SIRT1 and 2	Napper et al. (2007)
Cambinol	AD	SIRT1 IC50 = 56 μM SIRT2 IC50 = 59 μM	Inhibitor- SIRT1 and 2	Bilousova et al. (2018), Chowdhury et al. (2020)
Splitomicin	—	ySir2 IC50 = 60 μM SIRT1: No inhibition >500 μM	Inhibitor ySir2	Carafa et al. (2016)
Tenovin-1	—	IC_50_s not determined due to lack of water solubility	Inhibitor- SIRT1 and 2	Lain et al. (2008)
Tenovin-6	—	SIRT1 IC50 = 21 μM SIRT2 IC50 = 10 μM	Inhibitor- SIRT1, 2 and 3	Carafa et al. (2016)
Suramin	—	SIRT1 IC50 = 0.29 μM SIRT2 IC50 = 1.2 μM SIRT5 IC50 = 22 μM	Inhibitor- SIRT1,2 and 5	Schuetz et al. (2007), Trapp et al. (2007)
AK1/7	HD, PD	SIRT2 IC50 = 15.5 μM SIRT1/3 no inhibition >50 μM	SIRT2 inhibitor	Spires-Jones et al. (2012), Chen et al. (2015), Di Fruscia et al. (2015)
β-Lapachone	HD	—	SIRT1 activator	Shin et al. (2013), Lee et al. (2018)
AGK2	PD, HD	SIRT1 IC50 > 50 μM SIRT2 IC50 = 3.5 μM	SIRT2 inhibitor	Rumpf et al. (2015b), Chowdhury et al. (2020)
SirReal2	—	SIRT2 IC50 = 0.14–0.44 μM SIRT1 22% inhibition > 100 μM	SIRT2 inhibitor	Lancelot et al. (2013)
Cilostazol	AD	—	SIRT1 activator	Lee et al. (2014), Lee et al. (2019)
Salermide	—	SIRT1 IC50 = 43 μM SIRT2 IC50 = 25 μM	Inhibitor- SIRT1 and 2	Grozinger et al. (2001), Carafa et al. (2016)
ICL-SIRT078	PD	SIRT2 IC50 = 1.45 μM	SIRT2 inhibitor	Di Fruscia et al. (2015)
33i	AD	SIRT1 IC50 > 300 μM SIRT2 IC50 = 0.57 μM	SIRT2 inhibitor	Suzuki et al. (2012), Diaz-Perdigon et al. (2020)
24a	—	SIRT1 IC50 > 100 SIRT2 IC50 = 0.815 μM	SIRT2 inhibitor	Yang et al. (2018)
53	—	SIRT1 IC50 = 77.4 μM SIRT2 IC50 = 0.31 μM	SIRT2 inhibitor	Mellini et al. (2019)
6	—	SIRT2 IC50 = 1.74 μM SIRT1 14% inhibition = 50 μM	SIRT2 inhibitor	Li et al. (2014), Mellini et al. (2017)
36 (KPM-2)	—	SIRT1 IC50 = 1.56 μM SIRT2 IC50 = 0.055 μM	SIRT2 inhibitor	Mellini et al. (2019)
S1th 13	—	SIRT1 IC50 = 5.2 μM SIRT2 IC50 = no inhibition > 50 μM	SIRT1 inhibitor	Wössner et al. (2020)
γ-mangostin	AD	SIRT1 IC50 = 3.8 μM SIRT2 IC50 = 22.4 μM	SIRT2 inhibitor	Yeong et al. (2020)

Note: The *in vitro* IC_50_ reported for some of the inhibitors above was measured using assays that employ fluorophore-containing substrates that do not always reproduce deacetylation of the native substrate.

This review focuses on SIRT1 and SIRT2. Of these, SIRT1 is well-studied and the best understood mammalian sirtuin and SIRT2 is the most abundant sirtuin expressed in the brain. Both sirtuins are highly associated with neurodegenerative diseases. In this review we describe the structural aspects of both SIRT1 and SIRT2 to understand the mechanism of sirtuin modulation in neurodegenerative diseases, presenting activators and inhibitors that have either been confirmed or postulated to bind to the selectivity pocket, and provide an outlook regarding mechanistic investigations. The various technologies and methodologies that were used to design, synthesize, and validate potential drug molecules against SIRT1/2 activity are also elucidated. Further progress in our understanding of the mechanisms of sirtuin modulation by such compounds provides a basis for further drug development in the treatment of neurodegenerative diseases.

## SIRT1 and SIRT2 in ALZHEIMER’S Disease

About 1% of Alzheimer’s disease (AD) cases have a familial component, whereas 99% are sporadic and have a late-onset. The incidence and prevalence of AD increases exponentially with age, and in rare cases, AD can occur before the age of 60 (Alcaín et al., 2020). Familiar AD is associated with the autosomal dominant mutations in the amyloid precursor protein (APP), presenilin 1 (PS1), and presenilin 2 (PS2) genes as well as the ε4 allele of apolipoprotein E (Sherrington et al., 1995; Goate, 2006). Amyloid plaques and neurofibrillary tangles are histopathological hallmarks for AD (Serrano-Pozo et al., 2011). Hardy and Higgins were the first to publish findings on the accumulation of Aβ peptides in the brain parenchyma, indicating that this is a central event in the pathogenesis of AD (Hardy and Higgins, 1992). Under normal conditions, APP processing starts by α-secretase and later by the γ-secretase, forming a non-amylogenic peptide. APP can also be cleaved first by the β-secretase complex (BACE1) and later by γ-secretase, which produces the amyloid-beta (Aβ) peptide (Chow et al., 2010). Oligomeric Aβ induces glycogen synthase kinase-3beta (GSK-3β) expression in the brains of AD patients; this results in tau hyperphosphorylation and microtubule disruption originating the neurofibrillary tangles, impairment of memory and long-term potentiation (LTP), and neuronal apoptosis (DaRocha-Souto et al., 2012). Oxidative stress and mitochondrial dysfunction play an essential role in the early pathology of AD, and current therapeutic methods include novel usage of mitochondria-targeted antioxidants to scavenge free-radicals and improve the mitochondrial function (Oliver and Reddy, 2019). The protein quality control system and autophagic pathways are altered in AD, affecting the proteostasis balance (Llanos-González et al., 2020).

We identified SIRT1 as a premorbid and prodromal indicator of AD in 3xTg-AD mice (Torres-Lista et al., 2014). SIRT1 is expressed at lowered levels in the brains of patients with AD. In postmortem human brain samples from AD patients, the levels of SIRT1 in some brain regions were lower than those in the corresponding control group, and significant correlations were observed between the level of SIRT1 and Braak stage in these same regions of the brain (Lutz et al., 2014; Cao et al., 2018). The main risk factor for AD is age, and a decline in the serum concentration of SIRT1 in healthy individuals occurs during aging. In patients with AD and MCI, the decline was even more pronounced (Kumar et al., 2013).

**FIGURE 2:**
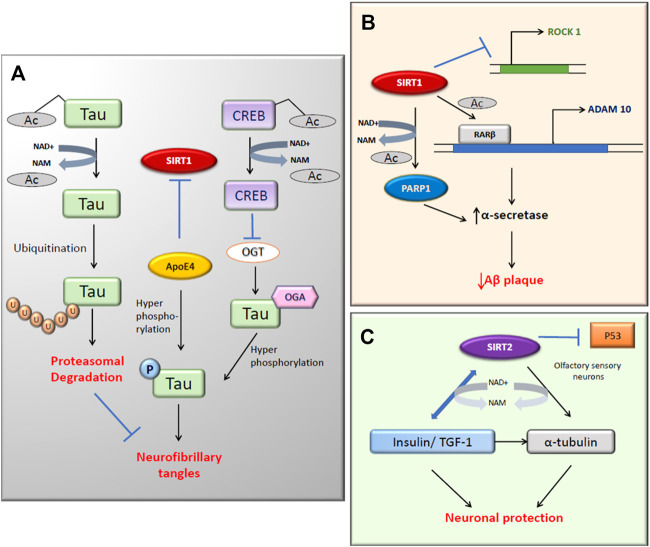
SIRT1 and SIRT2 activity in Alzheimer’s disease. **(A)** The deacetylation of tau protein by SIRT1 directs it to ubiquitination hence reduces neurofibrillary tangles (NFT). SIRT1 inhibition leads to the hyperphosphorylation of tau through increased activity of ApoE4 and OGT **(B)** The α-secretase activity increases with both the expression of ADAM10 and inhibition of deacetylated PARP1. **(C)** SIRT2 targets α-tubulin and Insulin/TGF-1 by deacetylation for neuronal protection.

SIRT1 regulates the non-amyloidogenic processing of APP through multiple targets (Qin et al., 2006). Poly (ADP-ribose) polymerases (PARPs) regulates the expression of SIRT1, transcription regulators, and amyloid precursor protein (APP) cleaving enzymes (Cantó et al., 2013). This oxidative stress induces PARP1 activation and contributes to mitochondrial dysfunction and cytotoxicity (Lu et al., 2019). The hyperacetylation of PGC-1α is also associated with the SIRT1 inhibition by PARP1, which can affect mitochondrial biogenesis. The activation of PARP1 depletes the NAD^+^ to inactivate SIRT1 and hence, the inhibition of PARP1, either by small molecules or by SIRT1 deacetylation, can, in turn, enhance the SIRT1 activity along with the transcription of enzymes involved in APP metabolism in Aβ cytotoxicity (Wencel et al., 2018; Figure 2A). In animal model studies, the inhibition of PARP1 showed reduced brain injury (Ma et al., 2012). As reported SIRT1 inhibits Rho kinase ROCK1 expression, which further influences the inhibition of the non-amyloidogenic α-secretase processing in brain cells (Figure 2A). By deacetylating the RARβ (Retinoic acid receptor β), SIRT1 upregulates the α-secretase ADAM10, and consequently, upregulates the non-amyloidogenic pathway. Inhibition of NF-κB down-regulates the expression of the β-secretase β-site APP-cleaving enzyme 1 (BACE1), reducing amyloidogenic APP processing (Figure 2A; Guo et al., 2016). Furthermore, SIRT1 also reduces Aβ generation by decreasing BACE1 transcription through PGC1-α and PPARγ deacetylation by direct interactions of SIRT1, PGC-1α and PPARγ activated proteins with the PPARγ responsive element/PPRE identified in the BACE1 promoter (Wang et al., 2013). The ADAM10 activation also leads to the Notch receptor cleavage, which targets the genes that are vital for memory learning and synaptic plasticity (Costa et al., 2005). However, specific pharmacological SIRT2 inhibition with AK7 increased non-amylogenic APP processing by α-secretase and reduced processing by BACE1, down-regulating its expression in the hippocampus and ameliorating the AD-associated pathology in 3xTg-AD and APP/PS1 mice (Wang Y. et al., 2020). Hence, SIRT1 and SIRT2 influence APP processing in opposing ways.

Hyperphosphorylated tau is associated with the reduced acetylation of cytoskeletal proteins (Mattson, 2003). SIRT1 deacetylates the hyperphosphorylated tau protein, which leaves them susceptible to ubiquitin ligases. This reduces the tau accumulation and neurofibrillary tangles (NFT) formation in cells (Figure 2B; Green et al., 2008). In this study, there was an increase in the level of acetylated α-tubulin with nicotinamide (NAM) treatment, which is a competitive inhibitor of sirtuins. SIRT1 deacetylates tau and reduces pathogenic tau spread in a mouse model of tauopathy (Min et al., 2018). In recent years, scientists have investigated the association between sirtuins and ApoE in AD (Cacabelos et al., 2019). According to the Bredesen group, ApoE4 reduces the ratio of neuroprotective SIRT1 to neurotoxic SIRT2 and also triggers the phosphorylation of tau and APP which leads to programmed cell death (Theendakara et al., 2013; Figure 2B). The O-GlcNAcylation of tau can negatively regulate the phosphorylation at Thr212 of tau, which can cause NFT formation (Liu et al., 2004). Tau phosphorylation is also regulated in an ApoE independent pathway in which CREB is involved (Lu et al., 2020). In AD, SIRT1 activation causes the deacetylated CREB to suppress the O-GlcNAc transferase (OGT) expression. This will, in turn, reduce the O-GlcNAcylation of tau and promotes the phosphorylation at Ser214 and Ser199, causing a reduction of NFT.

There are prominent shreds of evidence that prove the link between SIRT1 and AD (Bonda et al., 2011). In the last decade, many studies have focused on understanding the mechanism of action of SIRT1 in age-related neurodegenerative diseases (Rizzi and Roriz-Cruz, 2018). According to Anekonda et al., SIRT1 may deacetylate the AD-affected neurons in the nucleus, which prevents the apoptotic death of the neurons by repressing the p53 activity. The apoptotic activity of FOXO proteins is also suppressed, thus promoting neuronal survival. This presents an opportunity for novel therapeutic use (Anekonda and Reddy, 2006). Moreover, Aβ_25–35_-induced impairment of mitochondrial biogenesis in the primary hippocampal neurons is caused by the inhibition of the AMPK-SIRT1-PGC-1α pathway (Dong W. et al., 2016). The overexpression of the SIRT1 in the brain reduces central nervous system AD pathologies via activation of α-secretase directed cleavage of APP (Guarente, 2011). Researchers have identified that SIRT1 activation inhibits NF-κB signaling and reduces microglia-dependent Aβ toxicity (Chen et al., 2005). Sustained inflammation mediated by activated microglia is common to most neurologic disorders, and it has been reported that also SIRT2 overexpression inhibited microglia activation through NF-κB deacetylation (Pais et al., 2013). Decreased acetylation of SIRT1 substrates such as p53 and PGC-1α after resveratrol (RSV) treatment in the mouse hippocampus indicates the protective role of SIRT1 against neurodegeneration (Kim et al., 2007a). Severe impaired olfactory sensory functions in AD have led to the speculation that SIRT2 might be neuroprotective in olfactory sensory neurons (Anekonda and Reddy, 2006). According to De la Monte and Wands (2004), SIRT2 may also regulate the levels of insulin/IGF-1 in the cytoskeleton of AD brains (De La Monte and Wands, 2004).

SIRT2 was found to be the most abundant sirtuin in the brain, expressed exclusively in cytoplasmatic neurites and growth cones of postmitotic cells (Harting and Knöll, 2010). In AD, autophagy was found to be reduced. However, the inhibition of SIRT2 improves autophagy because SIRT2 is a tubulin deacetylase that regulates microtubule network acetylation. Microtubule disassembly impairs autophagy, whereas SIRT2 inhibition results in microtubule network reestablishment, facilitating axonal transport and fusion of autophagic vacuoles with lysosomes (Figure 2C; Silva et al., 2017). According to a meta-analysis study, the presence of SIRT2 polymorphism showed an association with AD in a European population (Wei et al., 2014). Though many scientists are trying to treat the cognitive impairment of AD, there are no drugs to prevent disease progression currently available. The expression of SIRT1 and SIRT2 during aging and the progression of AD differ considerably. SIRT1 mRNA expression levels were significantly decreased in both older people and AD patient groups compared to young people; however, the reverse is seen for SIRT2 (Wongchitrat et al., 2019). The oral administration of NAM, a non-selective SIRT1 and SIRT2 inhibitor but with lower IC50 to SIRT1 (Table 1), to 3xTg-AD mice, restored a cognitive deficit in these mice by reducing phosphorylated tau, and improved autophagy-lysosome procession in the hippocampus and cerebral cortex (Liu et al., 2013). Although the molecular mechanism is impossible to know because NAM inhibits both sirtuins, it seems that the inhibition of SIRT2, whose expression levels increase with age, may play an essential role in the improvement of cognitive abilities and the modulation of the most relevant molecular mechanisms in the progression of AD. Furthermore, SIRT1 were inversely correlated with insoluble hyperphosphorylated paired tau protein (Julien et al., 2009), the use of a specific SIRT2 inhibitor could increase the levels of SIRT1 indirectly and improve neuroprotection. Various SIRT1 enhancers such as resveratrol, SRT1460, and SRT1720 have been proven to reduce misfolding protein-induced neurotoxicity (Drygalski et al., 2018; Min et al., 2018).

**RSV** (3, 5, 4′-trihydroxy-trans-stilbene) is a polyphenol produced mainly in the skin and seeds of grapes. Initial studies showed that as a result of RSV treatment, cell survival in animal models increased by the stimulation of p53 deacetylation by SIRT1 (Howitz et al., 2003). RSV was found to extend the lifespan through the over-expression of SIRT1 in a mice model (Wood et al., 2004). RSV appeared to be protective against neurodegenerative diseases by activating SIRT1, AMPK, and PGC-1α (Baur et al., 2006; Lagouge et al., 2006; Dasgupta and Milbrandt, 2007; Rocha-González et al., 2008). RVS also showed a neuroprotective effect in a rat model of diabetes mellitus (DM) and concomitant AD that often coexist in patients (Ma et al., 2019). It delayed Wallerian degeneration by activating SIRT1 through dissociation from its inhibitor DBC1 in mouse models (Calliari et al., 2014). Long-term treatment by oral administration of *trans*-RSV on AβPP/PS1 mice (AD model) prevented memory loss (Porquet et al., 2014; Quadros Gomes et al., 2018). Furthermore, RSV treatment also increased the gene expression levels of IL1β and TNF in a mice model and influenced the inflammatory processes. **Piceatannol** (*trans*-3,4,3′,5′-tetrahydroxystilbene), a plant polyphenol, and a structural homolog of RSV showed a better protective effect compared to RSV on Aβ-induced PC12 neuronal cell death (Kim et al., 2007b; Karaman Mayack et al., 2020). Piceatannol reduced the ROS accumulation, thus decreasing the oxidative stress and attenuating apoptosis in Aβ-induced cells (Kim et al., 2008).

Another study explained the structure of SIRT1-RSV- p53- AMC (7-Amino-4-methylcoumarin) tagged peptide complex (PDB. 5BTR) and suggested that the stimulatory effect of RSV requires the presence of an N-terminal domain (NTD) and a catalytic domain (CD) of SIRT1 (Hubbard et al., 2013a; Milne et al., 2007), which is similar across all SIRT isoforms. The NTD of SIRT1 is not well studied; however, it is crucial to understand the mechanism of interaction between SIRT1 and RSV and how modulation of SIRT1 activity can lead to the development of novel pharmacological activators. The structure showed that two RSV molecules mediate the interaction between the AMC peptide and the NTD of SIRT1. These RSV molecules mainly help in tighter binding between SIRT1 and the peptide, hence stimulating of SIRT1 activity.

There are several ongoing clinical studies available to evaluate the benefit of RSV treatment in AD patients. According to a recent double-blind, placebo-controlled trial, oral intake of the high doses of RSV is safe, well-tolerated, and alters some AD biomarker trajectories (Turner et al., 2015). The treatment of 119 of these mild-moderate AD subjects for 52 weeks with RSV (up to 1 g by mouth twice daily) markedly reduced the levels of the cerebrospinal fluid (CSF) MMP9, a marker of blood-brain barrier damage, and as well as the CSF levels of Aβ_1-40_ and Aβ_1-42_. RSV also restricted cognitive decline in mini-mental status examination (MMSE) scores observed in the placebo group, suggesting that resveratrol may slow progressive cognitive and functional decline in mild to moderate AD subjects (Moussa et al., 2017). Ten subjects with a mild decline in cognition were randomized into an active grape formulation containing RVS arm or a placebo arm. These subjects consumed a formulation free of polyphenols for six months. The active formulation group was spared decline in regions of the brain that are significantly affected in the early stages of AD (Lee et al., 2017).

Previous research has shown that SIRT2 inhibition by the sulfobenzoic acid derivative **AK1,** affects the transcription of genes involved in cholesterol pathways in striatal neuronal cultures (Taylor et al., 2011). A study in rTg4510 mice (Frontotemporal dementia model mice) showed that the direct administration of AK1 in hippocampal regions prevented the neuronal loss by inhibition of SIRT2 (Spires-Jones et al., 2012). These results indicate the benefits of SIRT2 inhibitors in tau associated Alzheimer’s and Frontotemporal dementia diseases. SIRT2 inhibition by AK1 also confers neuroprotection via the downregulation of MAPK and FOXO3 pathways (She et al., 2018). Recent findings in AD cells proved that mitochondrial dysfunction could be managed by inhibiting SIRT2 expression by small molecules such as AK1 (Silva et al., 2017). The *in vitro* studies performed by Biella G *et al.* suggested that inhibition of SIRT2 by **AGK2** and **AK7** reduced the Aβ production in H4-SW neuroglioma cells and modified the APP proteolytic processing, leading to a reduction of soluble Aβ and an increase of soluble α-amyloid protein in two AD transgenic mouse models (3xTg-AD and APP23). This led to the improvement of cognitive performance in these mice. Furthermore, in the 3xTg-AD model, the authors found an increase of tau expression after AK7 treatment, while its phosphorylated form was undetectable (Biella et al., 2016). Both RSV and AGK2 reduces the reactive gliosis in AD models (Scuderi et al., 2014). Hence, the study supported the relevant mechanism for managing AD through inhibition of SIRT2. The treatment of AK7 in ER-stressed organotypic motoneuron cultures was also found to be neuroprotective (Romeo-Guitart et al., 2018). A naturally occurring β-amino acid, taurine, prevents Aβ_1-42_ induced mitochondrial dysfunction and neuronal death by activation of SIRT1 in SK-N-SH cells (Sun et al., 2014).

**33i** (2- {3-(3 fluorophenethyloxy) phenylamino} benzamide) is a selective inhibitor for SIRT2. According to a study, 33i compound modulates the glutamate receptor and neuroinflammation by SIRT2 inhibition in senescence-accelerated mouse prone-8 (SAMP8) model but only at an early stage. This could thus be a novel target to prevent age-related cognitive decline and neurodegeneration (Diaz-Perdigon et al., 2020).

Another set of oligomeric compounds called **procyanidins**, mixtures of catechin and epicatechin, could be effective in the treatment of AD. Some pathological features of AD such as extracellular amyloid deposits and neurofibrillary tangles have been observed to be attenuated by procyanidins. These compounds aided the enhancement of cognition and modulation of synaptic plasticity. Procyanidins upregulate the SIRT1 that stimulates CREB (cAMP response element-binding), which acts as a molecular switch from short to long term memory (Zhao et al., 2019). A new investigation has illustrated that procyanidins maintain cellular morphology and protect it from deformation and decrease apoptosis rates in PC12 cells induced by Aβ_25–35_ (Huang et al., 2018).

**Linagliptin** (marketed under trade names **Tradjenta)** is an inhibitor of dipeptidyl peptidase-4 (DPP-4) that has beneficial effects on impaired insulin signaling caused by Aβ in the SK-N-MC neuronal cells (Kornelius et al., 2015). Linagliptin activates the AMPK/SIRT1 pathway that further attenuates the mitochondrial dysfunction, and excessive ROS production caused due to aggregation of Aβ (Kornelius et al., 2015; Wiciński et al., 2019). This proves that DPP-4 inhibitors possess therapeutic potential for AD pathogenesis.

**3,4-Dihydroxyphenylethanol** (DOPET; hydroxytyrosol) is a biophenol that is present in olive oil, grape juice, and wine. It is an endogenous metabolite of dopamine that has central and peripheral neuroprotective effects (González-Santiago et al., 2006). It has been evaluated for its effects in an AD mouse model. The results showed that DOPET treatment reversed aberrations caused due to Aβ_1–42_ plus ibotenic acid intoxication such as dysregulation of SIRT1 and CREB associated protein expression levels in the hippocampus of mice. The increased SIRT1 activity elicits the α-secretase gene ADAM10 to enhance the clearance of neurotoxic Aβ peptide to protect the neuronal cells (Arunsundar et al., 2014). This reduced neurotoxic Aβ peptide decreases the tau aggregation and thereby has a potential therapeutic role in the treatment of AD.

**Cilostazol** (OPC-13013, 6-[4-(1-cyclohexyl-1H-tetrazol-5-yl) butoxy]-3,4-dihydro-2-[1H]-quinolinone) is a small molecule that reduced intracellular Aβ levels and phosphorylated tau in N2a cells. It also significantly enhanced learning and memory levels in a mice AD model (Park et al., 2011). A study proved that cilostazol suppressed the accumulations of FL-APP and Aβ by activating ADAM10 through the upregulation of SIRT1-coupled RARβ (Lee et al., 2014). According to research, cilostazol helps in the survival of N2a cells from Aβ-induced neurotoxicity by upregulating the autophagy machinery and its associated proteins. Inhibition of Aβ induced neurotoxicity was reversed by 3-methyladenine proving that the mechanism includes the induction of autophagy (Park et al., 2016). A clinical trial with 10 patients showed significant improvement in the MMSE score when donepezil with cilostazol was given in combination therapy (Arai and Takahashi, 2009). In a recent clinical trial, the administration of cilostazol in AD patients with white matter lesions showed improved cognitive function through increased glucose metabolism (Lee et al., 2019).

Finally, a first-in-class small molecule, apolipoprotein E4 (ApoE4)-targeted SIRT1 A03, showed SIRT1-enhancing effects in the hippocampus of 5XFAD-ApoE4 AD model mice improving their performance in cognitive tasks. The neurotoxic SIRT2 levels were not significantly affected (Campagna et al., 2018).

## SIRT1 and SIRT2 in Parkinson’s Disease

Parkinson’s disease (PD) is an age-related motor disorder caused by the loss of dopaminergic neurons in the substantia nigra of the midbrain (Kalia and Lang, 2015). It is associated with bradykinesia, postural instability, and neuronal loss. Another hallmark of PD is misfolded α-synuclein (α-Syn) protein aggregates, which form cytoplasmic inclusions called Lewy bodies (Spillantini et al., 1997; Krokidis, 2019).

SIRT1 and SIRT2 have contrasting effects in PD models. Previous studies suggest a genetic correlation between SIRT1 and PD based on the fact that the loss of SIRT1 or its mutations can lead to PD pathology, implying that SIRT1 is protective in function (Motyl et al., 2017). Though there are very few studies linking PD to SIRT1, α-Syn protein aggregation facilitates the dysregulation of mitochondrial function and reduces SIRT1 expression (Tang, 2016; Valdinocci et al., 2019). According to a study in animal and cell models, there is reciprocal regulation between SIRT1 and angiotensin-II in the substantia nigra. Manipulating this reciprocal regulation might be useful in neuroprotective treatment (Diaz-Ruiz et al., 2015).

**FIGURE 3:**
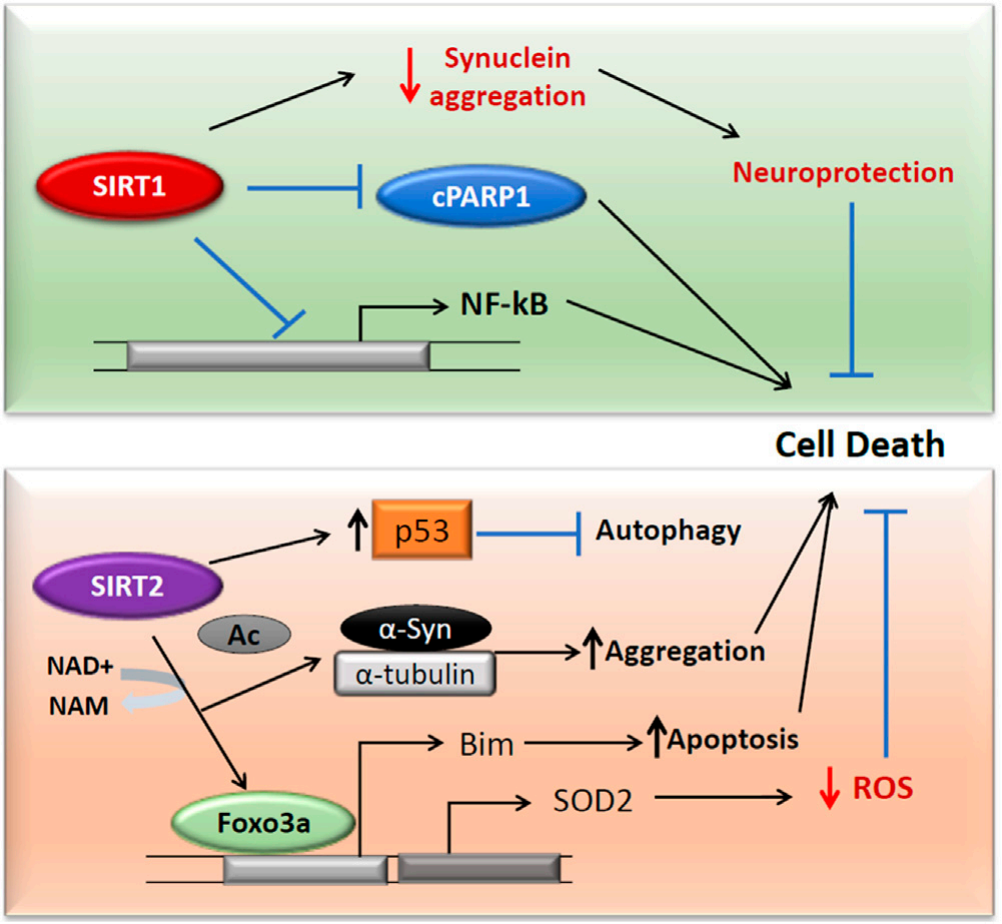
SIRT1 and SIRT2 activities in Parkinson’s disease. Both SIRT1 and SIRT2 act independently on cell death in Parkinson’s diseases. SIRT1 downregulates c-PARP and NF-κβ and reduces the protein aggregation in cells (Top). SIRT2 acts on FOXO3a to translate SOD2, which reduces the ROS in the cells (Bottom). The FOXO3a deacetylation also expresses pro-apoptotic Bim. SIRT2 can surge the protein aggregation through deacetylation of α-Syn and α-tubulin. The deacetylation of cytoplasmic p53 inhibits autophagy and can enhance the PD pathology.

In SH-SY5Y cells, SIRT1 has been shown to serve as a neuroprotectant by downregulating the expression of NF-κβ and cleaved PARP1 and reducing phospho-α-Syn aggregates (Figure 3; Singh et al., 2017a). In another study that aimed to elucidate the relationship between hypoxia-inducible factor 1α (HIF-1α) and SIRT1, SH-SY5Y cells were treated with methyl-4-phenylpyridinium (MPP^+^) to obtain a PD cell model. In this study the expression of SIRT1 was inhibited, however; the expression of HIF-1α and its target genes VEGFA (Vascular Endothelial Growth Factor A) and LDHA (Lactate dehydrogenase A) increased with MPP^+^ treatment. Furthermore, the silencing of SIRT1 increased the expression of HIF-1α, which suggested that SIRT1 was involved in the epigenetic regulation of HIF-1α (Dong S. Y. et al., 2016).

Nicotinamide phosphoribosyltransferase (NAMPT), the rate-limiting NAD biosynthetic enzyme, acts as a substrate for SIRT1 for NAD^+^ dependent deacetylation (Rongvaux et al., 2002). NAMPT plays a role in neuroprotection in PC12 cells against 6-hydroxydopamine-induced neurotoxicity (Zou et al., 2016). A dietary flavonol, fisetin (3,7,3′,4′-tetrahydroxyflavone) was demonstrated to protect the catecholaminergic PC12 cells via ROS scavenging and modulating the activity of SIRT1 and MAPK signaling (Yen et al., 2017).

SIRT2 has been reported to mediate α-Syn protein aggregation which also induces oxidative stress, in turn, leading to PD pathogenesis (de Oliveira et al., 2017; Liu et al., 2020; Singh et al., 2017b). SIRT2 by deacetylating FOXO3a has been shown to increase antioxidant defense mechanisms. It elevates the FOXO3a DNA binding, resulting in an increased expression of SOD2 (Wang et al., 2007). However, SIRT2 mediated FOXO3a deacetylation also increased the pro-apoptotic Bim (Wang et al., 2007; Figure 3). In PC12 cells, inhibition of SIRT2 by AGK2 has resulted in reduced ATP and cell death by necrosis (Nie et al., 2011). However, in contrast to these results, Nie et al. proved that SIRT2 inhibition protects PC12 cells from H_2_O_2_ induced toxicity and that its silencing reduced the levels of ROS following H_2_O_2_ treatment (Nie et al., 2014). SIRT2 can also promote cytoplasmic p53-dependent autophagy in a PD model (Sun et al., 2018). These results prove the contradictory roles of SIRT2 as both neuroprotective and neurotoxic in PD.

A recent study has demonstrated the neuroprotective role of **AGK2** in ischemic stroke through the downregulation of AKT/FOXO3a and MAPK pathways by selective SIRT2 inhibition in C57BL/6 mice (She et al., 2018). Additionally, the upregulated histone acetylation observed in PD was primarily due to the degenerating dopaminergic neurons and infiltrating activated microglia. The AGK2 treatment drastically reduced the activation of microglia and protected neurons from degeneration (Harrison et al., 2018). α-Syn acetylation regulates the distribution of α-Syn, which modulates aggregation and toxicity. SIRT2 deacetylates lysine 6, and 10 residues of α-Syn, and inhibition of SIRT2 modulates the levels of α-Syn acetylation, its aggregation, and autophagy (de Oliveira et al., 2017). According to a recent report, the downregulation of SIRT2 has a neuroprotective role in a middle cerebral artery occlusion (tMCAo) mouse model (Xie et al., 2017). The lipopolysaccharides-induced neuroinflammation and brain injury were also ameliorated by the inhibition of SIRT2 through AGK2 treatment in mice (Wang et al., 2016). Another substrate for SIRT2 is α-tubulin. SIRT2 overexpression inhibited neurite outgrowth in mouse hippocampal neurons by deacetylating α-tubulin and modifying microtubule dynamics, whereas SIRT2 knockdown cells resulted in a significant increase of neurite length (Pandithage et al., 2008). Recently it has been reported that γ-mangostin, a novel SIRT2 inhibitor displaying 6-fold selectivity against SIRT2 as compared to SIRT1 and SIRT3, promoted neurite outgrowth in an assay using the Neuro-2a cell line to evaluate the neurotrophic effects of a compound. Furthermore, the treatment of Neuro-2a cells with 2 μM of γ-mangostin showed a 40% increment in the number of differentiated cells after 48 h and may potentially be useful for the treatment of neurodegenerative diseases (Yeong et al., 2020). In the *Drosophila* model of PD, SIRT2 inhibitors showed neuroprotective activity in dopaminergic neurons by reducing α-Syn toxicity where fewer aggregates will be formed (Outeiro et al., 2007).

The various reports suggest that RSV has a therapeutic effect in PD treatment, and it has been shown to suppress the α-Syn-induced toxicity in SK-N-BE cells by activating SIRT1 (Albani et al., 2009). MPTP (1-methyl-4-phenyl-1,2,3,6-tetrahydropyridin), is a dopaminergic neurotoxin that induces most of the clinical features of PD. RSV improved PD phenotype by modulating MALAT1 and suppressing apoptosis of neurons in the MPTM induced PD model (Xia et al., 2019). Another line of evidence has shown that RSV ameliorates the pathological changes in MPTP treated mice through SIRT1 activation and light chain 3 (LC3) deacetylation. At the same time treatment with selisistat has reversed this effect of RSV by reducing LC3 deacetylation. Carboxamide selisistat was also found to affect the stable binding of the SIRT1-DBC1 complex in an acetylation-independent pathway (Hubbard et al., 2013b). 5,6,7,8- Tetrahydrobenzo[4,5] thieno[2,3-d]pyrimidin-4(3H)-one (ICL-SIRT07), a substrate competitive SIRT2 inhibitor which has been tested in MCF-7 breast cancer cells, and was found to increase acetylation of α-tubulin. Increased neuronal survival after the treatment of ICL-SIRT07 in a lactacystin-induced PD model of N27 cells has proved that SIRT2 inhibition could be a potential strategy for the treatment of PD (Di Fruscia et al., 2015). RSV administration to PD cells rescued the mitochondrial functions through the activation of AMPK/SIRT1/PGC-1α pathway. This emphasizes the therapeutic potentiality of RSV in the PD model (Ferretta et al., 2014).

The SIRT2 inhibitor **AK7** which is neuroprotective in HD (Chopra et al., 2012) was also evaluated for its effect in the PD model (Chen et al., 2015). It was found that AK7 is neuroprotective in models of PD but not amyotrophic lateral sclerosis and cerebral ischemia (Chen et al., 2015). However, from another recent study, the inhibition of SIRT2 by AK7 treatment or genetic depletion was found to be detrimental to motor neurons (Romeo-Guitart et al., 2018). In the MPTP induced PD mice model, the treatment of AK7 has improved behavior abnormalities, neurochemical deficits and redox dysfunction (Chen et al., 2015; Guan et al., 2016).

## SIRT1 and SIRT2 in Huntington’s Disease

Huntington’s disease (HD) is an autosomal dominant neurological disorder primarily associated with motor dysfunction. It mainly occurs due to an expanded CAG repeated sequence in the **huntingtin** (HTT) gene, which translates as a polyglutamine repeat in the protein product (Walker, 2007; La Spada, 2012). Mutant Htt proteins expressed ubiquitously in the peripheral and central nervous systems of HD patients form aggregates and these aggregates have been found to enter subcellular organelles, such as mitochondria interacting with the mitochondrial protein dynamin-related protein 1 (Drp1). The interaction causes excessive mitochondrial fragmentation and abnormal distribution, leading to selective synaptic degeneration (Reddy and Shirendeb, 2012). Despite the discovery of the mutant HTT gene more than two decades ago, the disease remains incurable and current treatment is focused on symptomatic therapy (Wyant et al., 2017).

**FIGURE 4:**
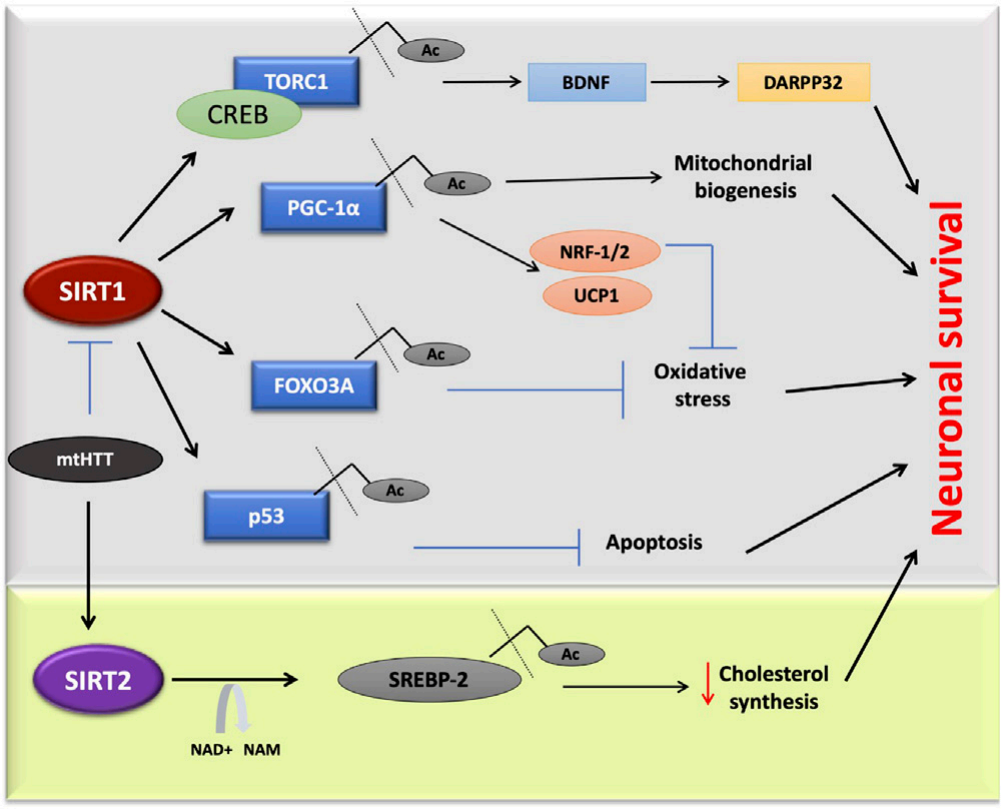
SIRT1 and SIRT2 in Huntington’s disease. Mutant huntington protein directly inhibits SIRT1 activity, affecting multiple pathways. In one of this pathway deacetylated TORC1 interacts with CREB, which is linked to DARPP32 and BDNF expression in neurons. Due to mutated HTT, the deacetylation of targets such as, TORC1, p53, FOXO3A, and PGC-1α will be inhibited that leads to cell death. Sterol biosynthesis also gets affected due to mutant. Inhibitors of SIRT2 found to decrease neurodegeneration in HD by the negative regulation of cholesterol biosynthesis.

Targeting SIRT1 and SIRT2 could be a potentially beneficial approach to the development of therapeutics for HD. SIRT1 is one of the most widely studied proteins that could serve as a potential protective agent against mutant HTT neurotoxicity. The mutation of the SIRT1 gene in the HD R6/2 model resulted in exacerbation of the disease. However, overexpression had the opposite result, wherein it reduced the Huntington aggregation. Genetically increasing the expression of SIRT1 served a neuroprotective role in transgenic mouse models of HD (Jeong et al., 2012). One of the mechanisms of action of SIRT1 is the deacetylation and activation of TORC1 (CREB regulated transcription coactivator1), accompanied by its binding with CREB to transcribe BDNF, a neuroprotective factor in HD (Jeong et al., 2012; Zuccato and Cattaneo, 2007). In the brains of HD mouse models (R6/2 and HdhQ150), SIRT1 activity is down-regulated where its phosphorylation status is affected due to abnormal expression of AMPK-α1 (Tulino et al., 2016). This regulatory mechanism of SIRT1 establishes a novel therapeutic strategy in the treatment of HD. HTT inclusions directly interact with SIRT1, causing an increase in the acetylation of its substrates such as FOXO3a and hence inhibiting the pro-survival effect of SIRT1 (Figure 4; Jiang et al., 2012).

There are other controversial observations regarding the effects of SIRT1 on HTT toxicity. SIRT1 overexpression showed decreased accumulation of HTT; however, in N171–82Q mice, SIRT1 did not affect HTT aggregation (Jiang et al., 2012). Other mechanisms of the SIRT1 action include the modulation of PGC-1α, a key player in the pathogenesis of HD, as well as the amelioration of mitochondrial dysfunction. These present promising avenues that could be utilized in the treatment of HD (Johri et al., 2013; Jodeiri Farshbaf and Ghaedi, 2017). One example of the different modes of SIRT1 activation is by the administration of nicotinamide riboside, a precursor of NAD^+^, which activates sirtuin enzymes also leading to the increased PGC-1α, SOD2, and mitochondrial reduced glutathione (Yang et al., 2007). In another study by Naia L et al., the mitochondrial function was completely restored by the treatment of RSV via activation of deacetylase activity. In contrast, NAM treatment increased the NAD^+^ levels and therefore increased H3 acetylation at lysine 9, in *in vitro* HD models (Naia et al., 2017).

Several studies have revealed that the significance of SIRT2 inhibition leads to a decline in neurodegeneration in animal and cellular models of disease (Luthi-Carter et al., 2010; Chen et al., 2015). According to these studies, manipulation of sterol biosynthesis mimicked SIRT2 inhibition, and the consequent metabolic effects of SIRT2 inhibition were sufficient to reduce HTT toxicity (Pallos et al., 2008; Luthi-Carter et al., 2010). In contrast to this, another study showed that a reduction in SIRT2 had no effect on the tubulin acetylation, cholesterol biosynthesis and failed to modulate disease progression in the R6/2 mouse model of HD (Bobrowska et al., 2012). It was concluded that SIRT2 inhibition does not alter the disease progression in the HID-R6/2 mouse model, and thus SIRT2 inhibition should not be considered as a therapeutic option for HD. More HD models need to be designed and examined to address this question.

**RSV** modulates cellular function by activating SIRT1. It also functions as a phytoestrogen to activate the estrogen receptors through which the mitochondrial electron transport chain is regulated (Albani et al., 2009; Gehm et al., 1997). In HD mitochondrial dysregulation plays a significant role in pathogenesis (Oliveira et al., 2006) and as mentioned, SIRT1 regulates the expression of PGC-1α, a key regulator of mitochondrial function and biogenesis (Figure 4; Nemoto et al., 2005b; Chaturvedi et al., 2009). RSV treatment in animal HD models has shown an improvement in cognitive and motor deficits (Kumar et al., 2006). An RSV injection of 25 mg/mouse/day for 75 days, was found to increase the PGC-1α level through SIRT1 deacetylation in the N171–82Q HD mice model (Ho et al., 2010). The small molecules SRT1720, SRT2183, and SRT1460 were found to be 1,000 times more potent than RSV in a type 2 diabetes disease model (Milne et al., 2007). The activator of SIRT1, **SRT2104** was found to attenuate brain atrophy, improved motor movement, and increased cell survival in the N171–82Q HD mice (Jiang et al., 2014). SRT2104 has been demonstrated as safe and has biological effects in both healthy and elderly populations of human phase I clinical trials (Libri et al., 2012; Hoffmann et al., 2013).

**Sesamin** and **sesamol** are also natural antioxidative compounds extracted from the sesame plant, which can act similar to RSV. Notably, pretreated neuroblastoma cell lines with sesamin and sesamol are potentially protected from oxidative stress through the activation of the SIRT1-SIRT3-FOXO3a pathway, which could be effective in HD treatment (Kumar et al., 2010; Ruankham et al., 2019). Another well-known natural compound serving as an activator of SIRT1 is **β-lapachone** (βL), which is isolated from the bark of the Lapacho tree. It is known to possess several beneficial effects in the treatment of diseases (Lee et al., 2015). The activation of SIRT1 by βL reduced the polyQ accumulation in the HD model and thus reduced its associated cytotoxicity (Shin et al., 2013). The treatment of R6/2 HD mice with βL significantly increased the SIRT1 expression, CREB phosphorylation, and PGC-1α deacetylation. Furthermore, oral administration of βL ameliorated the mitochondrial reactive oxygen species in HD mice (Lee et al., 2018). In cell-line studies, βL was found to induce the NQO1 expression, which further influences the redox homeostasis (Mahmoudinasab and Saadat, 2018; Siegel et al., 2018).

In the last decades, many SIRT1 and SIRT2 inhibitors have been identified by in silico screening. One among these is sirtinol, which acts on both proteins to inhibit deacetylation activity. In human cells expressing HTTEx1(97Q), sirtinol treatment increased the aggregation (Shin et al., 2013). The inhibitor sirtinol is beneficial in flies expressing mutant Htt (Pallos et al., 2008). Since sirtinol also inhibits SIRT2, it is not possible to exclude the notion that protection is due to inhibition of SIRT2, which has recently been observed to be protective in HD mouse models. NAM, the end product of sirtuins deacetylation activity, acts as a product inhibitor in the cell (Jackson et al., 2003). An initial *in vivo* study identified **NAM** as an effective suppressor of polyQ toxicity in spinocerebellar ataxia (Ghosh and Feany, 2004). Despite being an inhibitor of SIRT1, the treatment of B6.HD6/1 mice with 250 mg/kg/day NAM for 12 weeks was found to improve motor deficit by increasing the BDNF and PGC-1α levels (Hathorn et al., 2011). Though the NAM has a similar inhibitory effect as sirtinol, the scope is different. An acetylation screening assay has demonstrated the inhibition effect of both NAM and sirtinol in cells where acetylation sites upregulated by 12 and < 2% in the presence of NAM and sirtinol respectively (Schölz et al., 2015).

**Selisistat** (6-chloro-2,3,4,9-tetrahydro-1H-carbazole-1-carboxamide), a small molecule that selectively inhibits SIRT1/Sir2, was identified as a neuroprotective agent in *Drosophila*, mammalian, and mouse HD models (Smith et al., 2014). A study was conducted to examine the effects of genetic and pharmacological changes of SIRT1/Sir2 activity in the HD model. An exploratory double-blind, randomized clinical trial was performed in healthy volunteers as well as in HD patients to assess the safety, tolerability, and pharmacokinetics of selisistat. Based on the observations from this study, selisistat was identified as a candidate for further clinical efficacy studies in patients with HD (Süssmuth et al., 2015). Selisistat was first tested in humans to investigate the safety, pharmacokinetics, and pharmacogenomics of single and multiple doses in healthy male and female subjects. The double-blind, randomized, placebo-controlled study concluded that selisistat was safe and well-tolerated by healthy male and female subjects after single doses up to 600 mg and multiple doses up to 300 mg day (Westerberg et al., 2015). A randomized, double-blind placebo-controlled multicenter exploratory clinical trial with selisistat also established its safety and tolerability for the HD patients (Süssmuth et al., 2015). Selisistat has currently reached phase III clinical trials for the treatment of HD (Wössner et al., 2020). Sesame lignans (50 mg of sesamin/episesamin = 1/1) were also confirmed to be safe and tolerable in healthy subjects (Tomimori et al., 2013). However, it has been found that a βL analogue, ARQ 761, exerts toxicities such as anemia and methemoglobinaemia (Gerber et al., 2018).

A recent study identified that a novel thiazole-containing SIRT2 inhibitor **MIND4** has neuroprotective activity in brain slice and *Drosophila* models of HD. System biology approaches have inferred that MIND4 is a transcriptional inducer of the NRF2-mediated oxidative stress response (Quinti et al., 2016). According to previous studies, overexpression of NRF2 protects neurons from mutant HTT (Tsvetkov et al., 2013) and induces antioxidant effects in the brain (Ellrichmann et al., 2011).

Concerning HD, the modulators that stimulate SIRT1 deacetylation activity, as well as SIRT2 mutants with reduced activity, would significantly reduce the number of mHTT inclusions. Different SIRT2 inhibitors were investigated for their neuroprotective effect by examining decreased polyQ aggregation in HD models (Luthi-Carter et al., 2010). Structural modification of lead compounds **AK-7** and **C2-8** has resulted in the development of various analogues with improved SIRT2 inhibition potency, water-solubility, metabolic stability, and other desirable pharmacological properties. Khanfar and coworkers tested 176 analogues of sulfobenzoic acid for their effects on SIRT2 activity and, following this initial screen, a subset of bioactive compounds were evaluated for their tertiary polyglutamine aggregation assay in PC12 cells. The best substituents on the aromatic ring are cyano, acetyl, 1-hydroxyethyl, methylation (Khanfar et al., 2014). The sulfobenzoic acid derivatives such as **AK1** and AK7 were assessed for their efficacy and brain permeability (Taylor et al., 2011). A study proved that AK7 was more specific to SIRT2 compared to SIRT1 and SIRT3 and was found to improved neuronal survival, motor function, reduced brain atrophy, and the levels of aggregated mutant HTT in two genetic mouse models of HD (Taylor et al., 2011; Chopra et al., 2012). SIRT2 inhibition by AK1 downregulates the cholesterol synthesis by blocking the nuclear translocation of the sterol response element (SRE) binding protein 2 (SREBP-2) (Luthi-Carter et al., 2010; Naia and Rego, 2015).

## Insights Into the Role of Sirtuin Structures in Designing of New Modulators

The recent discoveries discussed above, emphasize the effective and beneficial roles of sirtuins in cancer and neurodegeneration. Although the importance of sirtuins in neurodegeneration is well understood, a lack of structural information has impeded the selective drug designing for SIRT1 and SIRT2. There are more promising data for the SIRT1 activators and SIRT2 inhibitors in the case of neurodegeneration. All isoforms of the sirtuins possess a conserved HDAC domain, and there exists an overlapping activity between them. SIRT2 was the first isoform structure to be uncovered in 2001 (Finnin et al., 2001). However, at present, SIRT1 is the more extensively studied isoform. Structural characterizations of a deleted SIRT1 construct have shed light on the modulator binding and key regulatory elements (Davenport et al., 2014; Cao et al., 2015; Dai et al., 2015; Zhao et al., 2016). There are more structures available for HDAC inhibitor complexes than SIRT1 activator complexes.

### Sirtuin Structure and Catalysis

**FIGURE 5:**
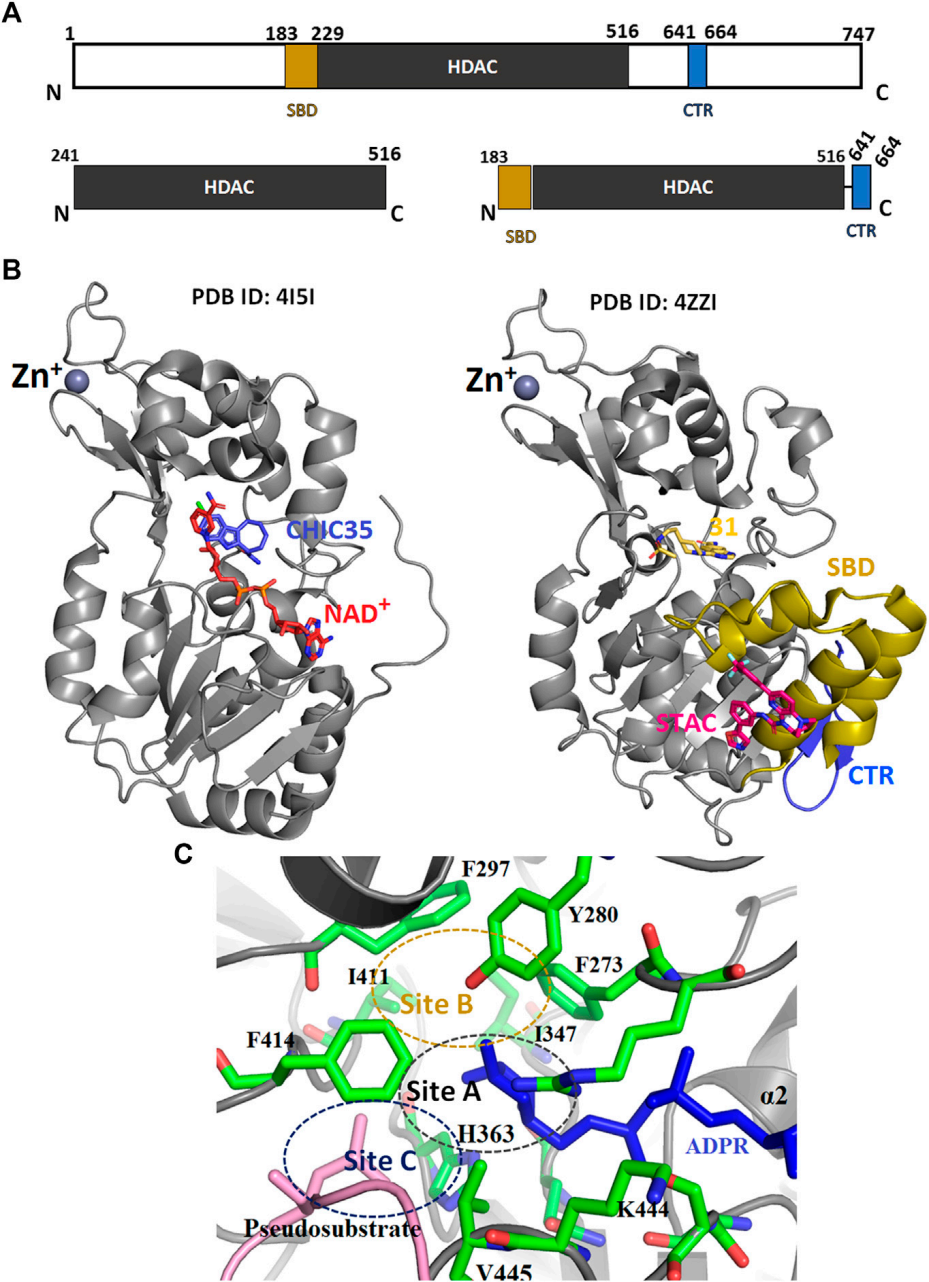
The tertiary structure of SIRT1. **(A)** Schematic representation of SIRT1 domain structure. **(B)** Crystal structure of HDAC domain with (PDB: 4ZZI) and without (PDB: 4I5I) extended NTD. **(C)** The catalytic sites **(A—C)** of HDAC domain. SBD-STAC binding domain: HDAC, Histone deacetylase: CTR, C-terminal regulatory segment.

Full-length SIRT1 consists of three major regions; the N-terminal domain (NTD) with three helices (183–229), the HDAC domain (229–516), and a β-hairpin from the C-terminal regulatory segment (CTR), which is also known as the region essential for SIRT1 activity (ESA) (Figures 5A,B **, Blue colored**) (Cao et al., 2015; Vaquero et al., 2004; Zhao et al., 2016). The NTD are also called sirtuin-activating compound (STACs) binding domains (SBD) (Figures 5A,B, **yellow-colored)**. This region binds to the activator AROS (Active Regulator of SIRT1), and are known as the allosteric site. A few studies have suggested that the rotation of SBD around the Arg234 residue would bring Glu230 close to Arg446, enabling their electrostatic interaction to stabilize the active conformation of enzymes and also bringing STACs close to the active site (Hubbard et al., 2013a). Additionally, the CTR segment was found to interact with the catalytic core to increase its stability (Kang et al., 2011).

**FIGURE 6:**
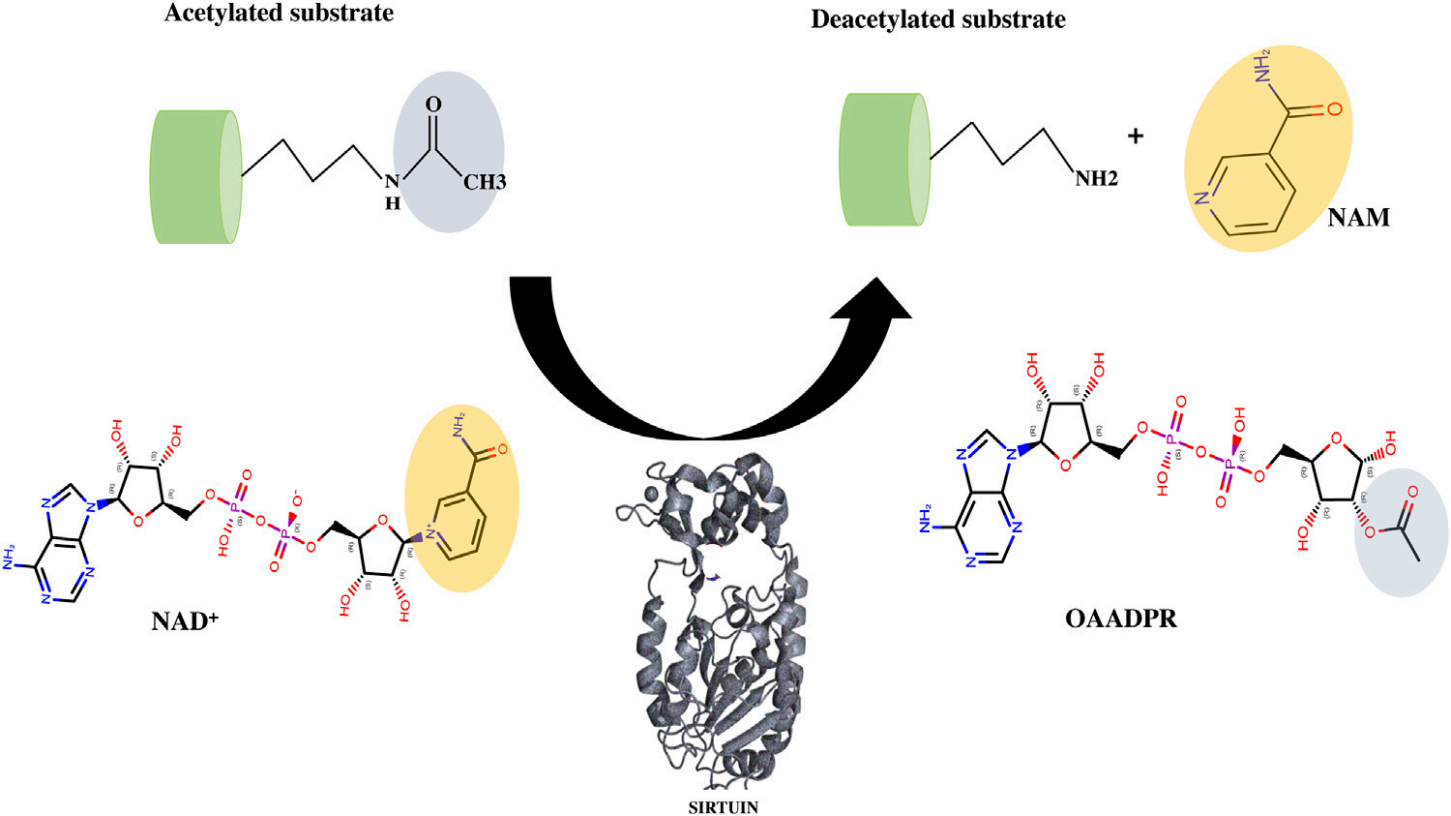
Deacetylation mechanism catalyzed by sirtuins. The cofactor NAD^+^ interacts with acyl lysine substrates to form an intermediate (not shown here) and releases the nicotinamide as a by-product. Further with the help of conserved catalytic His residue, sirtuin decomposes the intermediate to generate deacetylated lysine and the 2′-O-acyl ADPR (OAADPR).

Many SIRT1 and SIRT2 crystal structures of catalytic cores have revealed the conserved structure of the catalytic domain with the N- and C-terminal extensions varying in sequence and length (Moniot et al., 2012; Nguyen et al., 2013a). The overall structure of the HDAC domain of all isoforms is similar, with each in possession of a large Rossmann fold domain for NAD^+^ binding and a small domain that contains a zinc-binding ribbon module (362–419 residues) (Figure 5B). The substrate acetyl-lysine site is a hydrophobic tunnel between two domains. In all known structures the catalytic site can be divided into three sub-sites, namely, the NAD^+^ -binding region (site “A”), the inner hydrophobic cavity where nicotinamide (the product of NAD^+^, NAM) is released (site “B”), and the hydrophobic tunnel where the side chain of Kac binds (site “C”) (Padmanabhan et al., 2016; Figure 5C). During catalysis, NAD^+^ with its kinked conformation brings the C1′ of its ribose moiety into proximity for a nucleophilic attack by the carbonyl O atom of the acetyl-lysine that is inserted in a hydrophobic tunnel. On cleavage of nicotinamide and the formation of an alkylimidate complex, the acetyl group is transferred to the ADPR moiety, generating 2′-O-acetyl-ADPR and releasing the deacetylated lysine (Figure 6; Feldman et al., 2015).

Sirtuins adopt a different conformational state depending on the cofactor binding. SIRT1 also displays an open form in the absence of NAD^+^ and substrate, and a closed-form in the presence of cofactor and substrate (Davenport et al., 2014). Moniot et al. first demonstrated this conformational change in SIRT2 from an open conformation to a closed conformation upon ADPr and substrate binding, due to the small rotation in the Zn-binding domain (Moniot et al., 2013). The isoform two of SIRT2 was predicted to have an autoinhibitory C-terminal region that can partially block the NAD^+^ binding site and inhibit catalysis (Li et al., 2015). This naturally disordered region is responsible for the conformational change of the HDAC domain upon phosphorylation at Ser331 (Pandithage et al., 2008).

### Mechanism of Catalytic Activation

**FIGURE 7:**
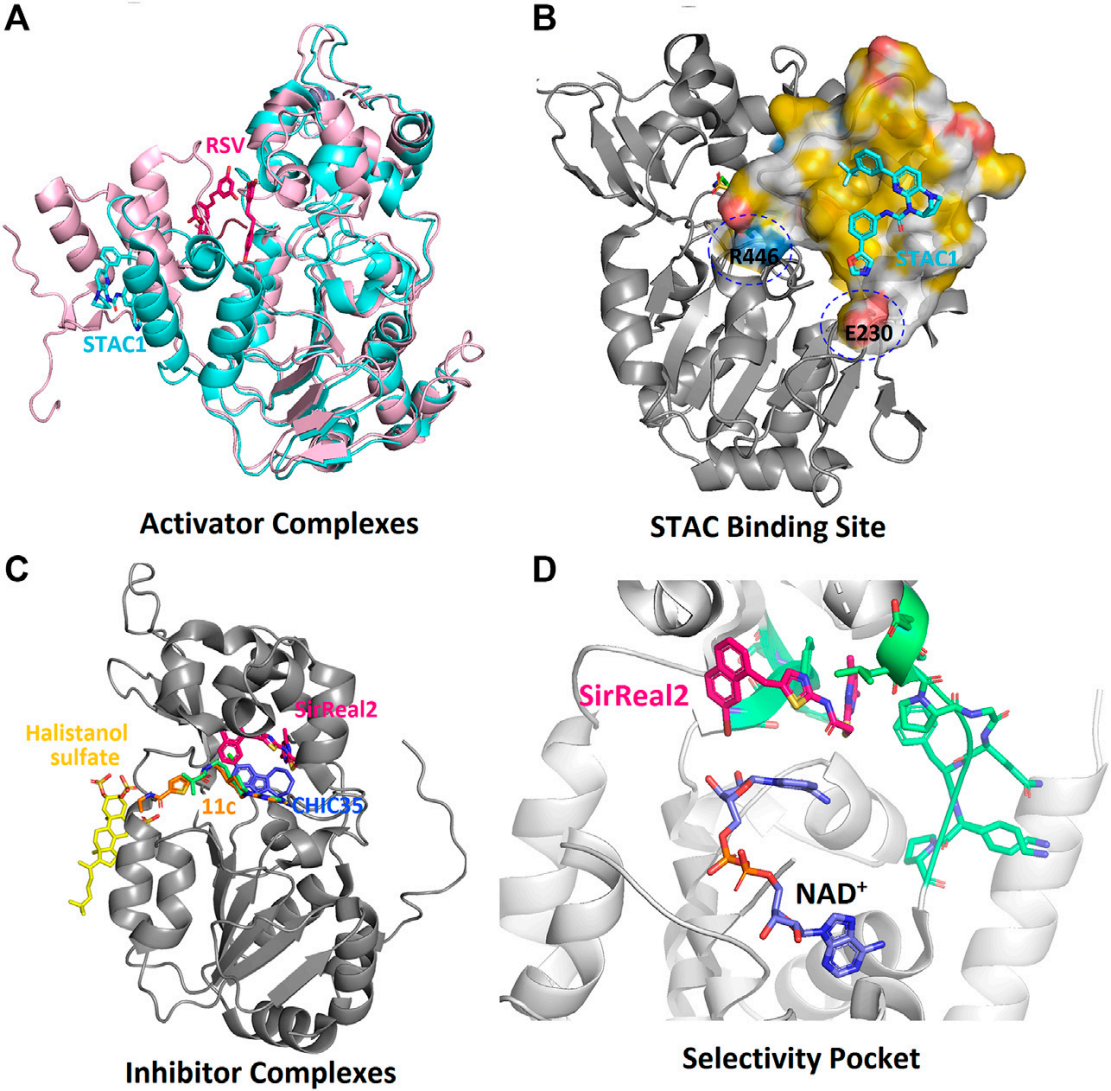
Complexes of sirtuins with the inhibiting and activating compounds (STACs). **(A)** Alignment of the two crystal structures of SIRT1-STAC complexes (PDB: 5BTR, 4ZZI). **(B)** Position of various inhibitors on HDAC domain. **(C)** The hydrophobic binding surface of SBD. **(D)**. The selectivity pocket (green) of SIRT2-HDAC domain responsible for the specific interactions of inhibitors.

According to Dai et al., the SIRT1 activation by STACs was reported to depend on the specific features of the fluorophore-labelled substrate in the deacetylation assay (Dai et al., 2010). In later years it was demonstrated that the hydrophobic motifs of the substrate were responsible for the STACs mediated activation (Hubbard et al., 2013a). Though there are multiple studies on SIRT1 and its activator, RSV, its mechanism of modulation by RSV is not well established. Despite its similarity to the RSV, Bromo-resveratrol acts as an inhibitor for SIRT1, and its binding site is not the same in the SIRT3-RSV complex (PDB: 4C78, Table 2; Nguyen et al., 2013a). According to this study, Bromo-resveratrol binds not only to the active site of the protein to inhibit substrate binding but also at the second site of allosteric modulation, where it could enhance the SIRT1 activity. The structure complex study of SIRT1 with RSV by Cao et al. analyzed RSV as the SIRT1-substrate interaction stabilizer (PDB:5BTR, Table 2; Cao et al., 2015; Hou et al., 2016). The study also emphasized the involvement of the NTD domain in substrate recognition of SIRT1. Though many studies have proved the SIRT1 activation by RSV through direct interaction, Cao et al. have verified this and concluded that the activation by RSV depends on the coumarin fractions of the polypeptide (Cao et al., 2015). This result corroborated several previous reports (Beher et al., 2009; Borra et al., 2005; Kaeberlein et al., 2005; Pacholec et al., 2010). In the SIRT1-RSV structure of 5BTR (Motta et al., 2004), the NTDs are positioned quite differently compared to the 4ZZI where SIRT1 is complexed with an activator (Dai et al., 2015; Figure 7A). According to Cao et al., the binding of the substrate with RSV bridges the NTD with p53 peptide. Based on this, the study concluded that a suitable molecule that mediates this interaction between the NTD and the substrate could further be used to activate the SIRT1 allosterically. However, Dai et al. have explained the activation profile of SIRT1 across different STACs and hypothesized that according to the mini-hSIRT1-STAC complex structures (PDB: 4ZZI, Table 2; Figure 7A), the NTD mediates STAC binding and activation of deacetylation, and the shallow hydrophobic surface depression of STAC-binding site matches the hydrophobic nature of the STACs (Figure 7B). On the other hand, interactions between the CTR and catalytic domain (Figure 5B, **grey-colored**) enhances the basal deacetylation activity (Dai et al., 2015). The same study suggested that the negative charge of Glu230 plays a role in stabilizing the activated conformation of hSIRT1 where it interacts with a positively charged residue in the activated state. The crystal structure of mini-hSIRT1, in both the studies, has shed light on the catalytic domain interaction with NTD and CTR. However, due to the difficulties in obtaining a full-length X-ray crystallographic structure of the SIRT1 enzyme, understanding of the molecular details of the STACs binding site and details about activated conformation remain elusive. Multiple computational analysis were performed to study the activation mechanism of sirtuin catalysis. An in-silico docking and molecular dynamics simulation of SIRT1 with a set of known compounds namely, metformin, ursolic acid (UA), and 1,4-dihydropyridines (1,4-DHPs), showed that these compounds are direct SIRT1-activating compounds (Cuyàs et al., 2018; Manna et al., 2018). The UA and 1,4-DHPs bind to the NTD and lead to the activation of SIRT1 through a protein–substrate interaction stabilization mechanism (Bakhtiari et al., 2018; Manna et al., 2018). The study on metformin delineates the putative binding modes to multiple pockets inside and outside of the catalytic domain, where it mimicked NAD^+^ boosters. According to a recent simulation study, the STACs that interact with Glu230 and Arg446 can stabilize the closed form of the SIRT1 (Liu and Yang, 2020). This proves that the NTD does indeed play a significant role in SIRT1 activation and hence, designing the NTD binding compounds should be a predominant strategy for the rational drug designing for associated diseases.

**TABLE 2:** The list of modulators found in RCSB PDB.

Name of the compound	Structure	Resolution
SIRT3-bromo-resveratrol (PDB: 4C78, 4C7B)	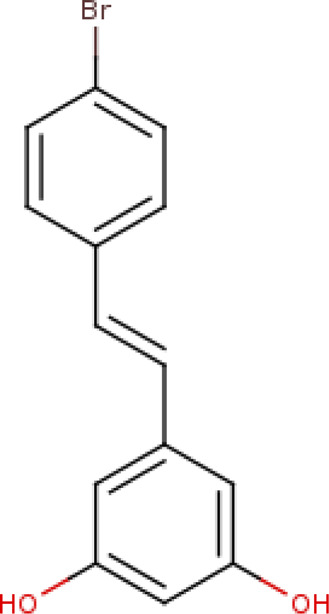	4C78–2.0 Å, 4C7B -2.1 Å Nguyen et al. (2013a)
Resveratrol SIRT1-RSV (PDB: 5BTR)	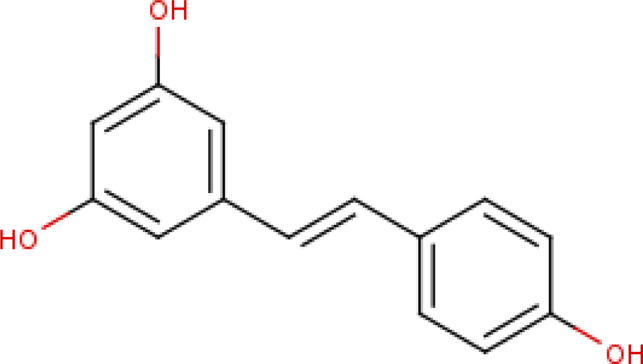	3.2 Å Cao et al. (2015)
(4S)-N-[3-(1,3-oxazol-5-yl)phenyl]-7-[3-(trifluoromethyl)phenyl]-3,4-dihydro-1,4-methanopyrido[2,3-b][1,4]diazepine-5(2H)-carboxamide SIRT1-STAC1(PDB: 4ZZH, 4ZZI, 4ZZJ)	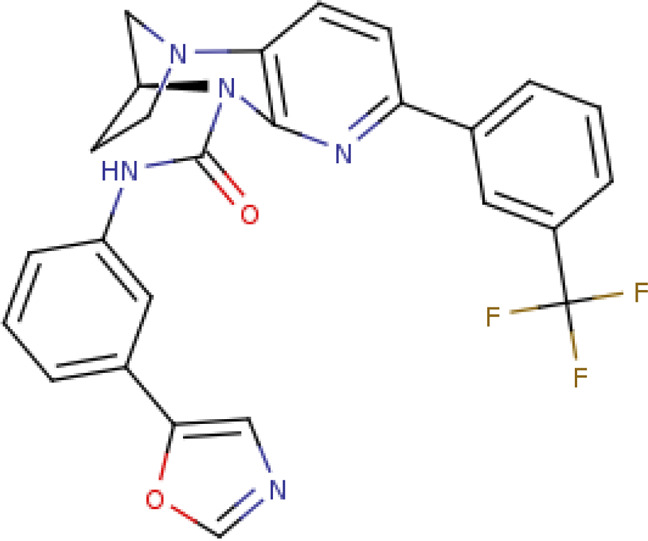	4ZZI-2.7 Å 4ZZJ-2.74 Å 4ZZH-3.1 Å Dai et al. (2015)
Pyridine-3-carboxamide Sir2Af2 -nicotinamide (PDB: 1YC2)	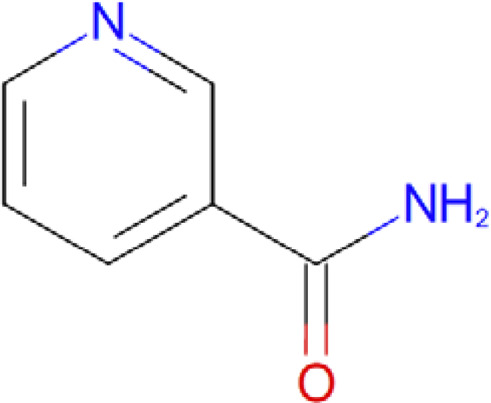	2.4 Å Avalos et al. (2005)
SIRT5-suramin (PDB: 2NYR)	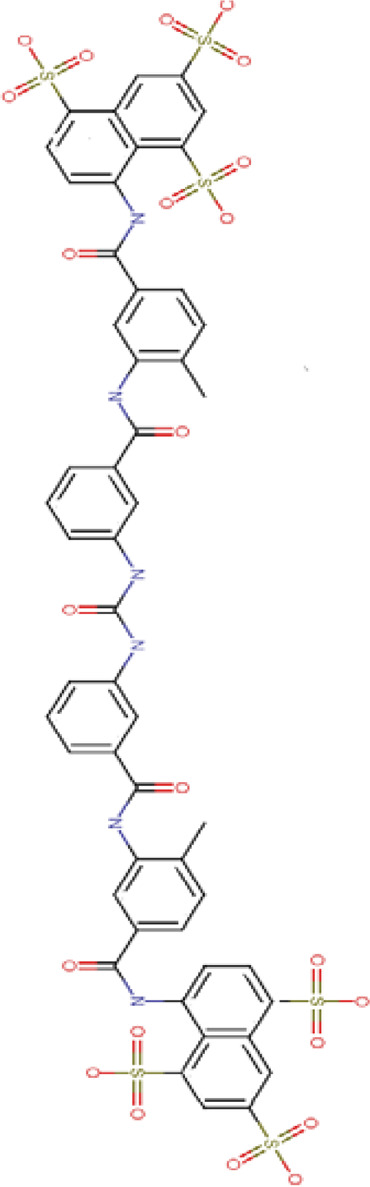	2.06 Å Schuetz et al. (2007)
6-Chloro-2,3,4,9-tetrahydro-1H-carbazole-1-carboxamide SIRT1-CHIC35 (PDB:4I5I) SIRT2-CHIC35 (PDB D:5D7Q)	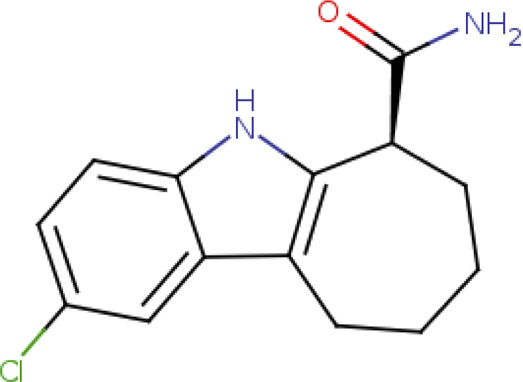	4I5I-2.5 Å Zhao et al. (2016) 5D7Q-2.01 Å Rumpf et al. (2015a)
4-(4-{2-[(methylsulfonyl)amino]ethyl}piperidin-1-yl)thieno[3,2-d]pyrimidine-6-carboxamide SIRT1-31 (PDB: 4ZZI) SIRT3-31 (PDB: 4JT9)	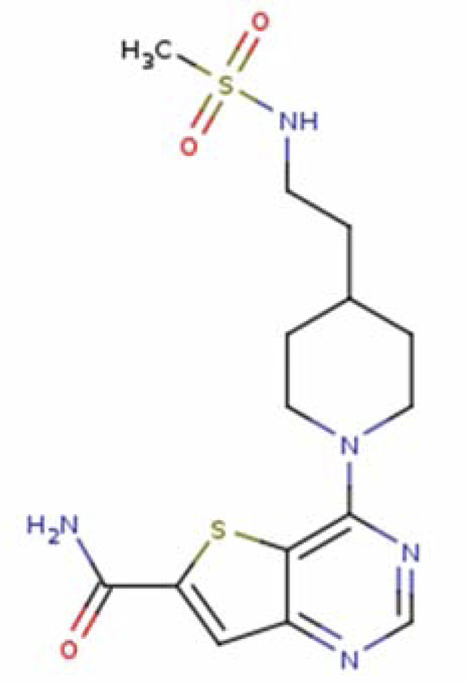	4ZZI -2.7 Å Dai et al. (2015) 4JT9- 2.2 Å Disch et al. (2013)
4-(4-{2-[(2,2-dimethylpropanoyl)amino]ethyl}piperidin- 1-yl)thieno[3,2-d]pyrimidine-6-carboxamide SIRT3-28 (PDB: 4JT8)	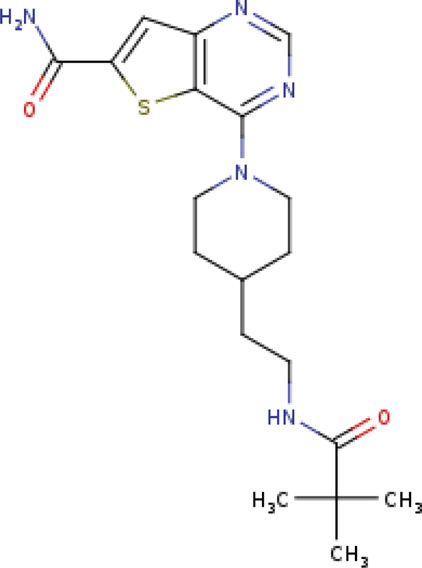	2.2 Å Disch et al. (2013)
N-{2-[1-(6-carbamoylthieno[3,2-d]pyrimidin- 4-yl)piperidin-4-yl]ethyl}-N′-ethylthiophene- 2,5-dicarboxamide SIRT3-11C (PDB: 4JSR)	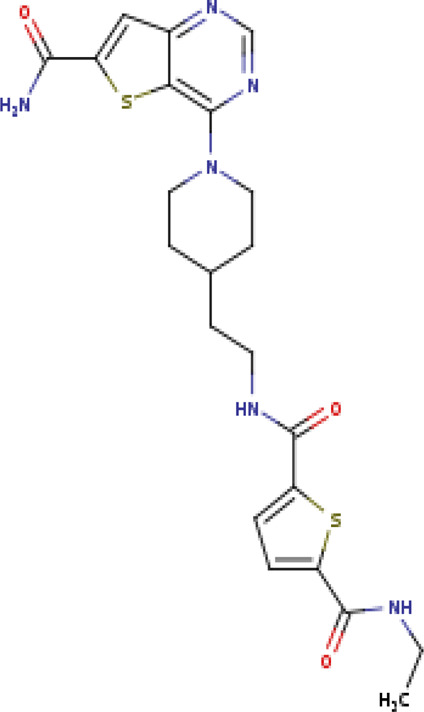	1.7 Å Disch et al. (2013)
SIRT2-SirReal2 (PDB: 4RMG, 4RMH)	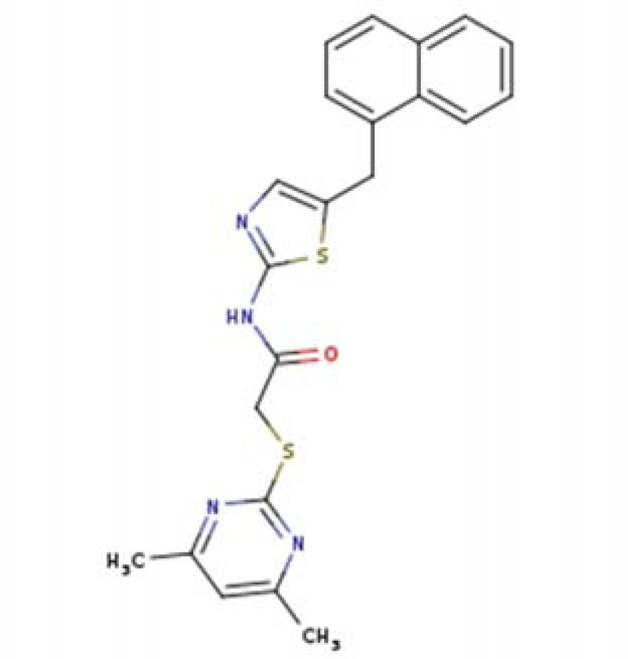	1.88 Å Rumpf et al. (2015b)
N-{5-[(7-bromonaphthalen-1-yl)methyl]-1,3-thiazol-2-yl}-2-[(4,6-dimethylpyrimidin-2-yl)sulfanyl]acetamide SIRT2-14a (PDB: 5DY4)	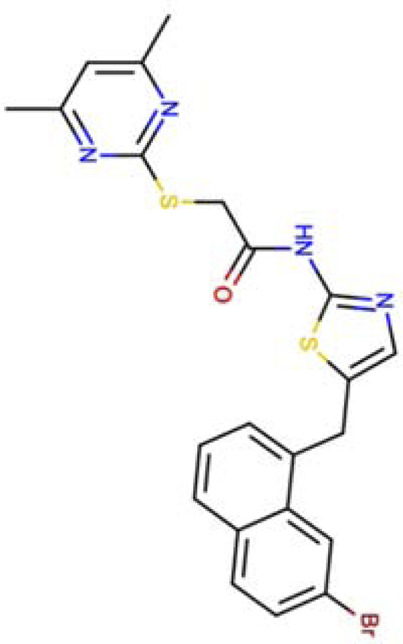	1.77 Å Schiedel et al. (2016)
(7∼{R})-7-[(3,5-dimethyl-1,2-oxazol-4-yl)methylamino]-3-[(4-methoxynaphthalen-1-yl)methyl]-5,6,7,8-tetrahydro-[1]benzothiolo[2,3-d]pyrimidin-4-one SIRT2-29c (PDB: 5MAT)	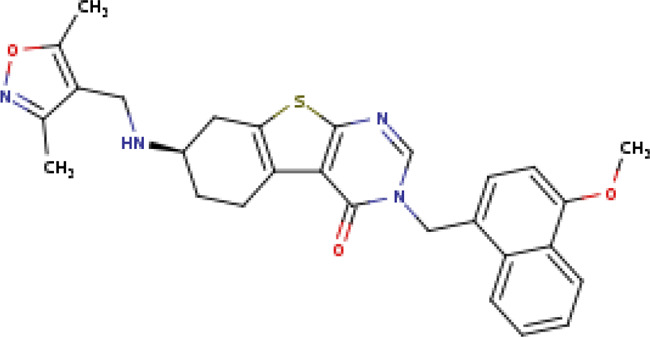	2.06 Å Sundriyal et al. (2017)
2-[[3-(2-phenylethoxy)phenyl]amino]benzamide SIRT2-6 (PDB: 5Y5N)	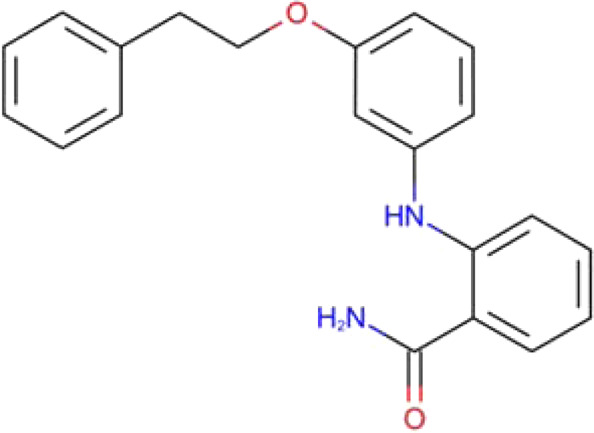	2.30 Å Mellini et al. (2017)
N-[4-[[3-[2-(4,6-dimethylpyrimidin-2-yl)sulfanylethanoylamino]phenyl]methoxy]phenyl]-1-methyl-pyrazole-4-carboxamide SIRT2-24a (PDB: 5YQO)	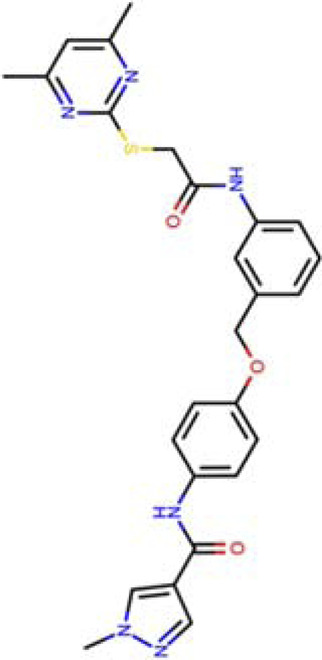	1.48 Å Yang et al. (2018)
[(2∼{S},3∼{S},5∼{S},6∼{S},8∼{S},9∼{S},10∼{R},13∼{R},14∼{R},17∼{R})-17-[(2∼{R})-6,6-dimethylheptan-2-yl]-10,13-dimethyl-2,3-disulfooxy-2,3,4,5,6,7,8,9,11,12,14,15,16,17-tetradecahydro-1∼{H}-cyclopenta[a]phenanthren-6-yl] hydrogen sulfate SIRT3-Halistanol sulfate (PDB: 5Y4H)	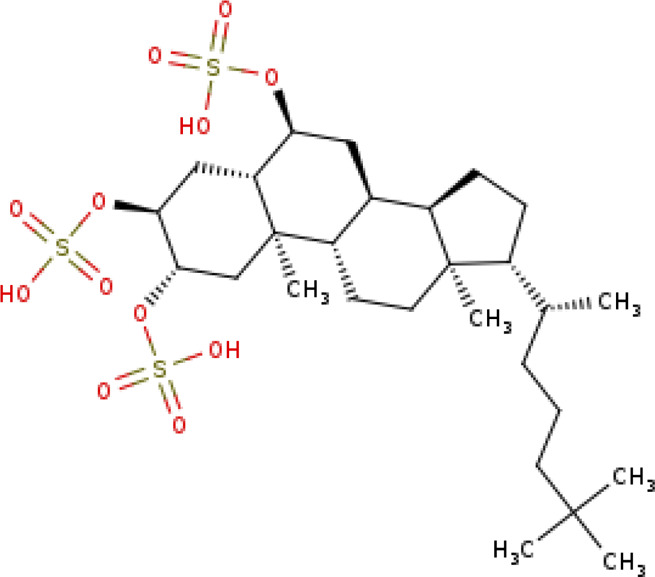	2.6 Å Nakamura et al. (2018)

### Mechanism of Catalytic Inhibition

Advancing current understanding of the distinct modes of SIRT1 and SIRT2 catalytic activity has become extremely challenging since there are no specific inhibitors that target only the protein of interest without influencing other family members. Structural information on sirtuin inhibition is still insufficient and there are very few reports on structures of sirtuins in complex with inhibitors (Nguyen et al., 2013b; Disch et al., 2013; Gertz et al., 2013; Rumpf et al., 2015a; Rumpf et al., 2015b; Zhao et al., 2016; Figure 7C).

**FIGURE 8:**
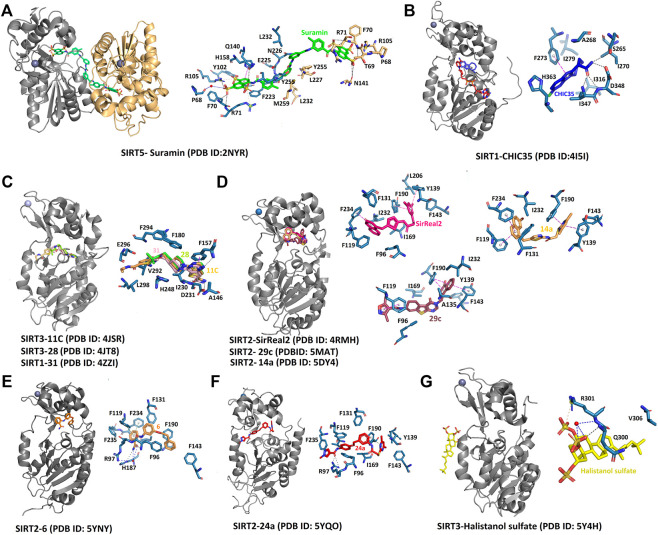
Complex structures of sirtuins with different inhibitors. The interaction of selective and non‐selective sirtuin inhibitors bound to the respective isoforms has been compared to understand the significance of specific residues. **(A)** Non‐specific inhibitor, suramin binds to substrate and cofactor binding sites. **(B)** Indole molecule Ex‐243 specifically binds to the SIRT1 HDAC domain. **(C)** The inhibitors with thieno [3,2‐d] pyrimidine‐6‐carboxamide scaffold specifically affects SIRT1 activity at the catalytic site. **(D‐F)** SIRT2 specific inhibitors bind to the selectivity pocket. **(G)** The only inhibitor that binds away from the catalytic core is Halistanol sulfate. Black, hydrogen bond; Blue, water‐mediated interaction; Pink, π– stacking.

The complex structure of the SIRT5 inhibitor, suramin complex (PDB: 2NYR), represents a binding site for an inhibitor that could block the binding of both the substrate and the cofactor (Schuetz et al., 2007). Suramin shows better inhibition for SIRT1/2 as compared to SIRT5 (IC50 = 22 μM, Table 1) and the binding site is in SIRT2 is similar to SIRT5 (Schuetz et al., 2007; Trapp et al., 2007). The natural non-competitive inhibitor, nicotinamide, inhibits the deacetylation through a base-exchange mechanism (Avalos et al., 2005; Jackson et al., 2003; Sauve et al., 2001; Sauve and Schramm, 2003). According to a study done by Avalos et al. (2005), the crystal structures of the complex archaeal Sir2Af2 (PDB: 1YC2, Table 2) and bacterial Sir2Tm (PDB: 1YC5) with nicotinamide have revealed the potential mechanism of sirtuin inhibitors. During the catalytic reaction, the bound nicotinamide can exist in either an entrapped or a reactive state that is interchangeable through a flipping mechanism. The study claims that the small molecules that bind to the catalytic pocket to prevent the productive conformation of NAD^+^ can serve as a competitive inhibitor. The non-competitive inhibitors act on the O-alkyl amidate intermediate, which leads to the base exchange. Suramin is one such non-competitive inhibitor. The crystal structure of the SIRT5-suramin complex contains one dimer in the asymmetric unit, and two suramin-linked monomers (PDB: 2NYR, Table 2; Figure 8A). Suramin mimics the interaction of nicotinamide in site-B and forms hydrogen bonds with the amino acids Tyr102, Arg105, Arg71, Arg141, and the main chain of Phe70. Tyr255 is located near the substrate-binding site and interacts with the urea carbonyl oxygen of the suramin. The molecular docking and dynamics simulation studies elucidate the importance of Arg97 and Glu167 interactions with the suramin and other inhibitors (Sakkiah et al., 2013). However, severe neurotoxicity and other systemic side effects are severe disadvantages to its use in treatment (Peltier and Russell, 2002).

The indole derivatives have been widely studied for the catalytic inhibition of SIRT1 protein (Huber et al., 2010; Manjula et al., 2019; Napper et al., 2007; Panathur et al., 2015; Zhao et al., 2016). In the study on the indole derivative, selisistat provided the NAD^+^ inhibition mechanism of catalysis (Gertz et al., 2013). Though there are no molecular differences in the binding sites of selisistat among sirtuin isoforms, the S-isomer of selisistat, Ex-243, interacts with the catalytic intermediate and dominates inhibition. This makes the indole compound more specific to SIRT1, as discussed in studies by Napper et al. Along with the Sir2Tm complex structures, the analysis of product formation suggests that Ex-243 inhibits the enzyme by stabilizing a sirtuin complex with the coproduct (Gertz et al., 2013). A crystal structure of SIRT1-NAD^+^ in complex with CHIC35 (PDB:4I5I, Table 2; Figure 8B; Zhao et al., 2016), an analogue of SIRT1 inhibitor selisistat, has defined a novel mechanism of deacetylation inhibition. Compound CHIC35 and NAD^+^ bind cooperatively and the inhibitor binding forces the extended NAD^+^ conformation which inhibits the substrate binding. In this structure, F273 forms π-stacking interaction with the indole of CHIC35 (Figure 8B). The AGK2 molecule is also expected to bind in the same site of SIRT2 (Outeiro et al., 2007). In 2015, Rumpf et al. obtained a complex of SIRT2-ADPR-indole by microseed matrix seeding (MMS), which also showed the same binding site for the indole derivative (Rumpf et al., 2015a). However, two indole molecules were found in the catalytic site for both selisistat and CHIC35 complexes.

Using Encoded library technology (ELT) and Structure-activity relationship (SAR), a new class of potent inhibitors based on a thieno [3,2-d] pyrimidine-6-carboxamide scaffold were discovered (Disch et al., 2013). As noticed in the SIRT3 complex crystal structure, the lead molecules 31 (PDB: 4JT9, Table 2), 28 (PDB: 4JT8, Table 2), and 11c (PDB: 4JSR, Table 2) are binding at the sites C and B of the catalytic site, forming a π–π stacking interaction with Phe157 through the thienopyrimidine core (Figure 8C). The complex structure of SIRT1 (PDB: 4ZZI, Table 2) with compound 31 also shows similar interactions, as seen in the SIRT3-31 complex (Dai et al., 2015). The binding of the inhibitor brings the small and large lobes together, leading to domain closure. The 6-carboxamide carbonyl of 11c forms hydrogen bonds with the protein backbone of Ala146, Ile230, and Asp231. The compounds lacking these hydrogen bonds were found to have reduced inhibitory activity. The ethyl piperidine of 11c with an extended conformation was bound at the hydrophobic cleft. Meanwhile, the arylamide is directed toward the substrate channel where it makes contact with Val292. The lack of this interaction also seems to affect the inhibitory activity of the compounds. However, a large portion of site-A was unoccupied, which could be further exploited efficiently to design inhibitors. All the potential lead molecules represented in the study showed low nanomolar inhibitory activity for SIRT1/2/3 and failed to have any isotypic specificity. The lack of isotypic specificity in the case of most HDAC inhibitors was later addressed in the interaction of SirReal (Sirtuin rearrangement ligands) with SIRT2. The slow-off rate and unique selectivity of these inhibitors made them a better option for therapeutics (Robaa et al., 2018).

The Rumpf group was successful in obtaining a crystal complex of SIRT2 with its selective inhibitor SirReal2, in which the binding of SirReal2 was found to lock the protein in its open form (Rumpf et al., 2015b). As seen in the crystal structure (PDB:4RMH, Table 2; Figure 8D), the naphthyl moiety of the inhibitor protrudes into the substrate-binding site, and the dimethylmercaptopyrimidine substituent (DMP) induces the formation of a ‘selectivity pocket’. This pocket is formed by the two loops (residues 136–144, residues 188–191) of the hinge region between the Rossmann fold domain and the zinc-binding domain (Figure 7D). At the selectivity pocket, the DMP forms a π–π stacking interaction with Tyr139 and Phe190 (Figure 8D). At the acetyl-lysine binding site the naphthyl moiety of SirReal2 forms van-der-Waals contacts with Phe131, Leu134, Ile169, Ile232, Val233 and π–π interaction with Phe234. The binding of SirReal2 adopts a conformation that almost perfectly complements the SIRT2, which is not possible in other sirtuins. This is because the residues that form the selectivity pocket are significantly different from those in SIRT2. The effect of SIrReal2 was also validated in an *in vivo* system, which proves that targeting this unexploited pocket may present a new strategy for selective sirtuin inhibitor design. However, SirReal2 has not been analyzed yet for its effect on neuronal survival in the diseased condition. Furthermore, the modification of SirReal compounds has yielded aminothiazole inhibitor **14a** with a similar binding site (PDB: 5DY4, Table 2; Figure 8D), but with improved SIRT2 inhibitory activity (Schiedel et al., 2016). The authors of this study also claimed that the quantitative structure-activity relationship (QSAR) model can rationalize the effect of designed compounds and can serve as the foundation for the development of the sirtuin rearranging ligands. The SAR analysis was utilized by Yang et al. to discover a SIRT2 inhibitor with nanomolar SIRT2 inhibitory activity (Yang et al., 2017). In 2017, Sundriyal et al., reported an extensive SAR study on the thienopyrimidinone SIRT2 inhibitor series and identified the key pharmacophoric elements associated with SIRT2 inhibition (Sundriyal et al., 2017). The crystal structure of the complex revealed the formation of the selectivity pocket in the SIRT2 active site. However, the binding was found to be in inverted in comparison to the SirReal2 complex (Figure 8D). Unlike SirReal2, **29c** induces the selectivity pocket through its 2-methoxy-naphthalen-1-yl ring forming π–π contacts with Phe119 and favourable hydrophobic interaction with the residues of the pocket (PDB: 5MAT, Table 2; Sundriyal et al., 2017). The positioning of the naphthyl ring structure has reoriented the side chain of the selectivity pocket residues Pro140, Phe190, Tyr139, Leu206, Lys210, and Phe214. The thienopyrimidinone ring of 29c occupies the region next to NAD^+^ binding site while making contacts with Ile93, Pro94, Phe96, and Ile1699 (Figure 8D). Another SAR and X-ray crystallographic study discovered a substrate-mimicking SIRT2 inhibitor that can inhibit both its deacetylase and demyristoylase activities (Nielsen et al., 2020).

A series of 2-anilinobenzamide derivatives have been synthesized and tested for their SIRT1-inhibitory activity in leukemic cells (Suzuki et al., 2009; Suzuki et al., 2006). The 3′-phenethyloxy-2-anilinobenzamide (**6**) derivatives represent a new class of SIRT2-isotype selective inhibitors (Li et al., 2014). The binding mode of this molecule, **6,** was determined by X-ray crystallography, and the structure revealed that the binding site is located at selectivity pocket (PDB:5Y5N, Table 2; Figure 8E; Mellini et al., 2017). The binding mode is similar to SirReal2-SIRT2 complex; however, compound 6 interacts with the lipophilic pocket at the site-C of the cofactor binding site. Furthermore, to target the substrate-binding site, a new compound **36 (KPM-2)** was designed, which was more specific to SIRT2 (Mellini et al., 2019). Thioamide **53**, a conjugate of diketopiperazine and 2-anilinobenzamide, is another inhibitor that is expected to bind at the substrate-binding site and the selectivity pocket (Mellini et al., 2019). Its structure-based drug design, using the SIRT2/**33a** complex, has resulted in an SIRT2 specific inhibitor **53**, which was tested for neurite outgrowth-inducing activity in Neuro-2a cells. These recent discoveries provide an insight into the specific inhibitor design for SIRT2.

A recent SAR study discovered a potent, selective SIRT2 inhibitor, which was validated in cancer cells (Yang et al., 2018). The *N*-(3-(phenoxymethyl)phenyl)acetamide derivative, **24a** was suggested to bind to the distinct hydrophobic pocket at the interface of the Rossmann fold domain and the zinc-binding domain of the SIRT2 with an IC50 value of 0.815 μM (Yang et al., 2018) (PDB:5YQO, Table 2; Figure 8F). A new SIRT2 selective inhibitor **TPN0_C7** that can specifically bind at this hydrophobic pocket was synthesized by Wang et al. (Wang X. et al., 2020). Interestingly, a molecular simulation study found the residues 87–125 and 290–320 to be more flexible. The inhibitors were shown to affect the stability of residues in the hydrophobic pocket. Hence this study opened up a new potential way to explore selective inhibitors.

There exist very few sirtuin inhibitors that interact with regions other than the catalytic site. Among such compounds are sulfate analogues, which inhibit sirtuins by binding to allosteric sites. Halistanol, a sulfate analogue (Table 2), is an antimicrobial, anti-HIV and anti-fouling compound. This compound showed SIRT1-3 inhibitory activity in HeLa and P388 cells (Nakamura et al., 2018). This inhibitory activity is suspected to be due to the detergent-like nature of the compound. However, the crystal structure suggests that the binding of the molecule induces allosteric structure changes in the SIRT3. Halistanol sulfate interacts with SIRT3 through hydrophobic interactions with Val306 and via hydrogen bonding with Gln300 and Arg301 (Figure 8G). To our knowledge, its neuroprotective effect on neurodegenerative diseases has not yet been studied.

Virtual screening, molecular docking and simulation studies have made a significant contribution to the discovery of novel sirtuin inhibitors (Padmanabhan et al., 2016; Eren et al., 2019). Based on previous studies, it is possible to design, synthesize and screen novel indole molecules that can potentially inhibit cofactor binding (Manjula et al., 2019). In recent years, scientists have made use of SirReal-based Proteolysis targeting chimaera (PROTAC) to inhibit SIRT2 (Schiedel et al., 2018). **Chroman-4-one** and **Chromone** derivatives were also tested for SIRT2 inhibition, and 6,8-dibromo-2-pentylchroman-4-one was proved to be a potential inhibitor with an IC50 of 1.5 μM (Fridén-Saxin et al., 2012; Karaman Mayack et al., 2020). By using previously known thiomyristoyl (**TM**) lysine peptides, a new soluble inhibitor glucose-conjugated TM was synthesized and crystallized in complex with SIRT2 (Hong et al., 2019). However, as the N- and C- terminal ends of the TM structure did not contribute toward any significant binding, a new set of soluble inhibitors were designed.

Multiple docking studies and biochemical assays have shown that the indole molecules mainly bind at the catalytic site to inhibit the deacetylating activity of the sirtuins (Napper et al., 2007; Suenkel et al., 2013; Pulla et al., 2014; Panathur et al., 2015). Other than indole molecules, benzofuran (Xu et al., 2017), 2-anilinobenzamide analogues (Suzuki et al., 2012), 1,4-bispiperazinecarbodithioic acid methyl esters series (Zheng et al., 2016), chromanone derivative (Fridén-Saxin et al., 2012), oxycoumarin and diphenyl derivatives (Padmanabhan et al., 2016), pyrazolone and isoxazol-5-one cambinol analogues (Medda et al., 2009; Mahajan et al., 2014), 6,7-dichloro-2-oxindole series (Huber et al., 2010; Verçoza et al., 2017) and a series of thieno [3,2-d] pyrimidine-6- carboxamides (Cui et al., 2014) were reported to have high inhibition for sirtuin activity. Most of these inhibitors have not been tested on NDD models. There are very few studies that have focused on the specificity of SIRT1 inhibition and a very recent molecular dynamic simulation study revealed a novel selectivity pocket of SIRT1 (Singh et al., 2020). The study came up with the novel amino acid coupled SIRT1 selective inhibitor. A phenyl thiocyanate was also found to be a selective inhibitor for SIRT1, as identified in a recent study (Wössner et al., 2020). As SIRT1 inhibition was focused on cancer treatment, the inhibitory thiocyanates (S1th) were tested for antiproliferative activity, inhibition of migration, and colony formation as well as hyperacetylation of Sirt1 targets p53 and H3 in cervical cancer cells (HeLa). The inhibitor was thought to engage with the Asn465 and Asp272 through hydrogen bonds and Cys482 via covalent interaction. As mentioned earlier, the phytochemical, γ-mangostin also acted selectively on SIRT2, and the docking analysis showed the interaction of the compound with the catalytic domain. The inhibitor was found to bind between the Rossman fold, and the zinc-binding domain interacts with Gly 86, Gly 261, Glu288, Asp95, and Asn286 (Yeong et al., 2020).

## Conclusion and Future Perspectives

Mammalian sirtuins are a class of critical factors that have been shown to play important roles in the homeostasis of tissues and organs, as well as in the regulation of numerous cellular processes. However, the complexity of sirtuin activity, which is heavily dependent on cellular context and experimental conditions, increases the difficulties in determining how best to modulate them therapeutically. In general, the downregulation of sirtuins is associated with age-related diseases, oxidative stress and mitochondrial dysfunction. However, sirtuins can also show completely opposite effects. For instance, activation of SIRT1 is neuroprotective in AD, PD, and HD.

Contrary to this, the inhibition of SIRT2 is neuroprotective in AD, PD, and HD. Hence, tissue-specific modulation of sirtuins is necessary in order to have a net neuroprotective effect in neurodegenerative diseases. As all isoforms of sirtuins possess a conserved HDAC domain, it is crucial to develop modulatory molecules specific for each type of sirtuin. This review has detailed the effects of SIRT1/2 modulators in neurodegenerative diseases and also elucidated the structural aspects in order to understand their binding site residues. A survey of the literature indicates that SIRT1/2 proteins could serve as potential therapeutic targets in the treatment of cancer and neurodegenerative diseases. Thus, the discovery and development of sirtuin modulators are of prime importance. Currently, there are multiple pharmacological agents used to regulate sirtuins. These have been demonstrated as safe and effective in the case of neurodegenerative diseases. However, further investigation is required to understand the mechanism of activation of HDAC activity. In silico analysis has been effective in the design of drugs and developing potential ligands against neurological disorders, however, additional rigorous analysis is required.

Multiple activators of SIRT1 are being tested for a variety of pathologies. Natural STACs (piceatannol and RSV), as well as synthetic activators (SRT2104, 1,4-DHP derivatives and SRT1720), are being tested for their specificity and efficacy. Since obtaining a full-length SIRT1 crystal structure has proven to be very difficult, there exist very limited complex structures to deduce the structural mechanism of SIRT1 activation at present. Though the SBD and CTR regions are involved in forming contacts with activators, their binding residues in SIRT1 are yet to be discovered. A ligand can allosterically activate SIRT1 when it bridges NTD with the substrate or bind to the hydrophobic motifs of NTD to stabilize the active state of the SIRT1. As a well-known HDAC activator, RSV has shown multiple health benefits in clinical trials. However, the role of RSV as a sirtuin activator is still under debate.

The currently available inhibitors mainly target the catalytic site of the HDAC domain at Sites A, B, and C, where they either bind and inhibit the substrate and cofactor binding or allosterically change the structure of the protein. The inhibitors common to all isoforms compete either for the cofactor or the substrate-binding site. However, the non-competitive inhibitors act on an intermediate molecule of the catalysis. Both SIRT1 and SIRT2 expressions are tissue-specific and vary in different physiological conditions and diseases. The primary challenge in designing a sirtuin modulating drug is to obtain an isotype-specific molecule that can be effectively used for specific diseases. Of late, extensive usage of the ELT and SAR methodologies has led to the discovery of specific inhibitors such as SirReals for SIRT2. These techniques also shed light on unsuccessful trials of virtual screening to discover specific sirtuin modulators. This specificity is mainly due to the selectivity pocket formed by the two loops between the Rossmann fold and the Zn-binding domain. Furthermore, to discover highly sensitive and specific inhibitors, modulators were designed to target the lipophilic pocket of site C along with the selectivity pocket. According to a recent study, the hydrophobic pocket at the interface between the Rossmann fold and the Zn-binding domain could also be a new target to design selective inhibitors for SIRT2. Allosteric modulators such as SirReals could potentially show great value in the future of drug design, and multiple drug preparation trials have taken place over the past ten years that have specifically targeted sirtuins. Despite the many thousands of new investigations into drugs very few have successfully progressed to clinical trials.

## Author Contributions

RM and FJA devised the idea of the study. RM, KA, and FJA contributed to the literature search. RM and KA designed and drafted the figures. RM prepared the initial draft of the manuscript. KA and FJA contributed to, reviewed and approved the final draft of the paper.

## Funding

This study was supported by grants from MINECO (SAF 2016–79311-R), Consejería de Educación, Cultura y Deportes (SBPLY/19/180501/000245), and UCLM (2020-GRIN-29101) to FJA.

## Conflict of Interest

The authors declare that the research was conducted in the absence of any commercial or financial relationships that could be construed as a potential conflict of interest.
